# Determinants of Academics' Job Satisfaction: Empirical Evidence from Private Universities in Bangladesh

**DOI:** 10.1371/journal.pone.0117834

**Published:** 2015-02-20

**Authors:** Abdul Kadar Muhammad Masum, Md. Abul Kalam Azad, Loo-See Beh

**Affiliations:** 1 Department of Administrative Studies & Politics, Faculty of Economics & Administration, University of Malaya, Kuala Lumpur, Malaysia; 2 Department of Business Administration, International Islamic University Chittagong, Chittagong, Bangladesh; 3 Department of Applied Statistics, Faculty of Economics & Administration, University of Malaya, Kuala Lumpur, Malaysia; TNO, NETHERLANDS

## Abstract

The job satisfaction of academics is related to a number of variables of complex function such as demographic characters, the work itself, pay, work responsibilities, variety of tasks, promotional opportunities, relationship with co-workers and others. Academics may be simultaneously satisfied with some facets of the job and dissatisfied with others. This paper aims at determining the influential factors that contribute to the enhancement or reduction of academics’ job satisfaction among private universities in Bangladesh with special reference to Dhaka, the capital city of Bangladesh. A total of 346 respondents are considered from ten private universities using non-probability sampling. A pre-tested and closed-ended questionnaire using a seven-point Likert scale is used for data collection. In this study, descriptive statistics, Pearson product moment correlation, multiple regression, and factor analysis are exercised as statistical tools. A conceptual model of job satisfaction is developed and applied for academics’ job satisfaction. The results reveal that compensation package, supervisory support, job security, training and development opportunities, team cohesion, career growth, working conditions, and organizational culture and policies are positively associated with the academics’ job satisfaction. Amongst them, three factors stood out as significant contributors for job satisfaction of academics i.e. compensation package, job security, and working conditions. Therefore, the management of private universities should focus their effort on these areas of human resource management for maintaining academics’ job satisfaction and employee retention. The study will be useful for university management in improving overall job satisfaction as it suggests some strategies for employee satisfaction practices.

## Introduction

Universities create and cultivate knowledge for building a modern world where leaders are groomed to lead the nation with social justice. There are many factors that should be considered especially with regards to the welfare of human resource in achieving the mission and vision of the universities. One of these factors is job satisfaction, as it is important for retaining employees. In Bangladesh, private universities are enjoying the superior position to public universities in terms of the number of students’ enrolment and establishment growth [1]. Unfortunately, the turnover of academics at most of these private universities is critical in Bangladesh [2]. An article written by Jalil [3], published in the national daily newspaper of Bangladesh, focused on the turnover of academics because of job dissatisfaction and noticeably supported earlier research. The author stated that academics’ turnover per year was only 2% to 3% for public universities; while it was 16% to 17% and sometimes the rate was even higher for some private universities in Bangladesh. Because job satisfaction is one of the main factors affecting the tenure of employees, it is important to consider job satisfaction in establishing an employee retention plan. Satisfied employees tend to be more creative and committed in the organizations [4]. Therefore, job satisfaction must be considered important in managing university academics. This article aims to provide a modest contribution to the understanding of factors and mechanisms that influence academics’ job satisfaction in private universities in Bangladesh.

An employee bears a positive view for his work responsibilities because of his job satisfaction. Definitions of job satisfaction given vary between authors. Job satisfaction is defined as the emotional status and attitude of an employee towards his work [5]. Klassen, Usher and Bong [6] identified academics as a distinct sample subset of human resources because of their dissimilar working environments in comparison to typical employees of an organization. Moreover, academics have personal and professional interests with regard to their universities, including pressure to pursue excellence, to make the right decisions regarding the research agenda and course load, and ensure steadiness between work life and family life [7]. As a consequence of job dissatisfaction of academics, their performance will be decreased and they will be incapable to contribute to education sector. For this reason, this study focuses on disclosing the factors of job satisfaction of academics.

The purpose of this study is to examine the factors associated with job satisfaction amongst Bangladesh private university academics with reference to an extended version of Gibson, Gibson and Ingold [8] conceptual framework. The conceptual model of academics’ job satisfaction developed in this paper is based on work-related factors, such as human resource and organizational factors. Research for investigating the associations between job satisfaction and human resource management practices is inadequate in the context of higher education institutions in Bangladesh. The main thrust of this paper is in classifying the most influential factors that affect academics’ job satisfaction for their retention at the higher education level. By knowing the influential factors, only then appropriate policy responses at the institutional level can be initiated. Some leading private universities in Bangladesh are tested using this proposed model. The paper also proposes strategies for improving employee satisfaction practices in private universities. Results of the study can provide a guideline for university management in providing an effective job satisfaction and employee retention policy. This paper is organized as follows: the literature of academics’ job satisfaction is briefly discussed followed by factors influencing academics’ job satisfaction in private universities. Finally, limitations of this study and future research directions including managerial implications are discussed.

### Literature review

Numerous studies have been conducted on job satisfaction of typical organizations’ employees. However, only a limited number of studies have focused on academics’ job satisfaction as a subject of study. Klassen, Usher and Bong [6] identified promotion, pay, supervisory support, team cohesion and the job requirements itself as the prime factors of job satisfaction. In addition, Maertz and Griffeth [9] in a theoretical exposition reported eight motivational factors for job satisfaction which comprises of competitive salary, job autonomy, good supervision and interpersonal relationships, training and development opportunities, better working conditions, and job security. In addition, some researchers identified organizational culture as another factor of job satisfaction [10–13]. In a study on job satisfaction of university academics in Uganda, Ssesanga and Garrett [14] employed nine determinants to measure academics’ job satisfaction. These include teaching, research, remuneration, supervision, opportunities for promotion, co-worker’s behavior, working conditions, governance, and the job itself. Whereas in a study of private universities in China, Chen, Yang, Shiau and Wang [15] explored six determinants that influence the academics’ job satisfaction in private universities of China, namely organization vision, result feedback and motivation, management system, working condition, pay, and benefits. For the present study, we selected eight independent variables, namely compensation package, supervisory support, job security, training and development opportunities, team cohesion, career growth, working conditions, and organizational culture and policies to investigate the influence of these variables on the dependent variable—academics’ job satisfaction.

Next, the key job satisfaction dimensions are described in detail in the following paragraphs. Among the dimensions of job satisfaction, compensation package is regarded as the most influential factor of job satisfaction for employees of any organization [16]. The management of private universities often provides various pay packages including special pay premiums, incentives, bonuses, etc. for employee retention. Pay packages and professional development were identified as the most significant factors of job satisfaction at Massachusetts Institute of Technology (MIT) [17]. Similarly, Noordin and Jusoff [18] found salary to be one of the most important conditions amongst Malaysian academic staff members for their job satisfaction. In more detailed study by Weiler [19] found that pay packages is a significant motivational factor of job satisfaction for junior academics but not for senior academics at the Associate Professor and Professor levels. Ehrenberg, Kasper and Rees [20] reported that the compensation package is the only significant factor for junior faculty members such as Lecturers and Senior Lecturers. On the other hand, Chew and Chan [21] pointed that compensation package is not the only factor that helps retain talents in organizations. In contrast with the aforementioned studies, Khatri, Fern and Budhwar [22] opined that the compensation package is not a significant factor in job satisfaction in Asian context with reference to Singapore. However, this study was in a general organizational context and not in the academic setting. It is worth mentioning that Singapore is an advanced country compared to Bangladesh, a third world country, and the findings of the study may not hold water in Bangladesh.

Supervisory support refers to employees’ awareness regarding the point to which supervisor value their contributions and is concerned about their welfare [23]. There exists an affirmative relationship between supervision support and job satisfaction for academic staffs of higher education [24–26]. According to organizational support theory, employee performance increased because of supervisor’s supportive behavior that enhances employee dedication, and contribution to attain organizational goals. It also increases employees’ intention to stay in organization [27]. Some researchers stated that supervisors have interpersonal relationship with their subordinates, and this relationship may persuade employees’ job satisfaction in the job place [28–30]. On the contrary, Ashraf and Joarder [31] in the context of Bangladesh found insignificant influence of supervisory support on the rating of attitude towards academics’ job satisfaction and job retention in Bangladesh private universities. Similarly, Billah [32], in a separate study on Bangladesh commercial banks, also did not find any significant association between job satisfaction and supervisory support. Supervisory support will be furthered explored in this study with a larger sample of academics and universities.

Job security refers to the demonstration by the organization that an employee has only a small chance of becoming unemployed, and in return, the employee will give commitment to the organization [33, 34]. This factor is very important for job satisfaction and supports the theory of the norm of reciprocity [35] and social exchange theory [36]. Ashford, Lee and Bobko [37] found that job satisfaction and organizational commitment of employees declined because of job insecurity although Chughtai and Zafar [38] argued that job satisfaction is only slightly affected by job security. In the context of job security, the staffs of private organizations are less satisfied compared with those of public organizations [24]. Most of the private universities, with little exception, are suffering from high levels of academic staff turnover due to job early on in Bangladesh [39]. According to Jalil [3] and Joarder and Sharif [1], they early faculty turnover in private universities in Bangladeshis between 12% and 18%. Thus, job security is included for its negative impact on job satisfaction in this study.

Training and development is an essential function of human resource management for supporting employees in acquiring the skills and new knowledge needed for the desired performance in a competitive environment [40]. Training and development hasa potential effect on academics’ job satisfaction [34, 41]. Saleem, Shahid and Naseem [42] stated that job satisfaction and employee productivity are enhanced by training and development programs since by these programs employees are equipped with the skills they need to do their job efficiently. Haines III, Jalette and Larose [43], in a study on Canadian Non Governmental sector, under scored that employees’ turnover decreased due to training and development experienced by the employees throughout their career to enhance their skills. In contrast, Way [44] did not find a significant relationship between training and employee turnover within the US small business sector. Due to the contradictory results and the lack of explicit evidence of the relationship between job satisfaction and training in an academic setting, further investigation is required.

Currently, working with peers or team cohesion is encouraged for project assignments, because it creates opportunities for social interaction between employees [15, 45]. An employee who is a member of a team has more obligations to the team’s efforts and to the organization. Viswesvaran, Deshpande and Joseph [46] stated that there is a highly positive relationship between co-workers’ attitudes and job satisfaction. In corroboration, Kreitner, Kinicki and Buelens [47] stated that responsive and helpful attributes of co-workers improve job satisfaction of employees. Moreover, Schmalenberg and Kramer [48] reported that employees who do not receive assistance from co-workers are more likely to experience dissatisfaction in their jobs. Ashraf and Joarder [31] also argued that working with peers has important effects on academics’ job satisfaction among the private universities of Bangladesh, in contrast to most academics of public universities, who are overly occupied with national politics [24]. Because the existing literature shows a lack of clear proof regarding the influence of team cohesion or co-worker assistance in relation to job satisfaction, this variable is included in this study for further investigation.

Opportunities for promotion or career growth lead to employee retention in organizations. In recent studies, researchers identified career growth as a significant factor for job satisfaction of academics [24, 31, 49]. Similarly, Rahman and Parveen [50] reported that academics of both public and private universities in Bangladesh show dissatisfaction when they lack the opportunity of fair promotion. It is important to note that the rules and policies for promotion in all the public universities are alike and structured by the government. In contrast, in private universities, it varies from one university to another, as most of them do not have structured rules and policies for promotion. This is also evident in Bangladesh as there are vast dissimilarities and biasness in promotion practices among the private universities. As such, this variable is incorporated into this study.

Employees appreciate a friendly or responsive workplace. Usually, a friendly workplace does not involve a large amount of investment and expenditure, but it requires time and sympathetic thoughtfulness on the part of top management. Schmalenberg and Kramer [48] empirically proved a positive relationship between employee job satisfaction and healthy working conditions. Good working conditions reduce employee turnover and induce a lower degree of job stress. Undesirable outcome on employees’ dedication may be created if they are dissatisfied with the working conditions and consequently it may affect turnover decision [49]. A study on workers of GrameenPhone, the largest telecom company in Bangladesh, identified working atmosphere or working condition as the most significant factor of job satisfaction for the employees of that company. This factor was also identified as a dominant factor for employees’ decision to leave or to remain with the organization in a university setting [31]. Conversely, Joarder and Sharif [1] found an opposite result than the earlier studies. They disagreed that working conditions is a significant factor for increased job satisfaction for academics of higher educational institutes in Bangladesh. Herzberg [51] also argued that employees’ commitment may not increase with working conditions. Previous literature shows contradictory results on the association between job satisfaction and working conditions. Hence further investigation is required with regard to this variable.

Organizational culture refers to a set of value systems and propositions around performing the operations of an organization [2]. Huang and Chi [52] described that organizational culture and policy encourage employees to be motivated and consistent in their commitments. Consequently, it leads to an increased organizational performance. Some researchers [10, 53] concluded that organizational culture and policy have a positive influence on the job satisfaction of the employees. In contrast, Johnson [54] argued that some elements of organizational culture might not be significantly related to employees’ job satisfaction. Similarly, Navaie-Waliser, Lincoln, Karuturi and Reisch [55] asserted that job satisfaction of employees is not affected by organizational culture and policy. The inconsistent relationship between job satisfaction and organizational culture and policy needs further investigation to inform the relationship. Therefore, the variable of organizational culture and policy is incorporated into the present study.

The literature on factors influencing job satisfaction reveals mixed results. In this study, we examine eight key dimensions of job satisfaction to determine the most influential factors for job satisfaction amongst academics of private universities in Bangladesh.

### Conceptual framework

**Fig 1:**
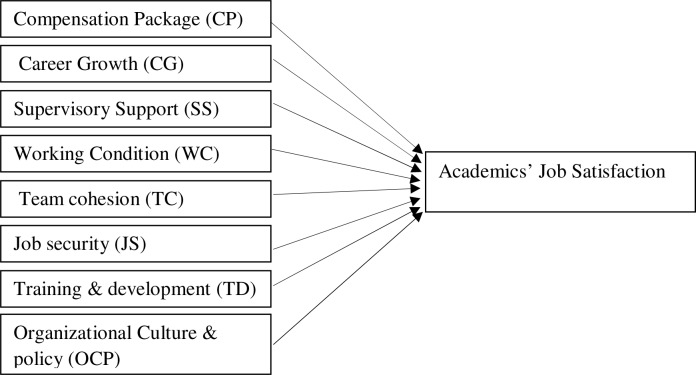
Conceptual model for academics’ job satisfaction at private universities. This figure indicates that the eight HR and organizational factors examined influence the job satisfaction of academics at private universities in Bangladesh.

Employees’ job satisfaction can be categorized in two types based on their feeling regarding their jobs- the job facet satisfaction and the global job satisfaction. The former refers to employees' overall feelings about their jobs while the latter refers to feelings about specific job facets, such as benefits, salary, and the quality of interaction with one's co-workers [56]. This study is concerned with the job-related satisfaction. There are some human resource (HR) and organizational factors that affect the job satisfaction of academics. Among them, we examined eight independent factors for academics’ job satisfaction. The selected factors comprise of compensation package [16–18], supervisory support [29, 24–26], job security [3, 33, 34, 39], training and development opportunities [34, 41–44], team cohesion [24, 31, 47, 48], career growth [41, 49, 50], working condition [1, 31, 48], and organizational culture and policy [1, 10, 52, 54]. Fig. 1 shows a conceptual model of academics’ job satisfaction which is an extension of the existing model by Gibson, Gibson and Ingold [8]. They identified five dimensions of job satisfaction that include salary or compensation packages, supervision or supervisory support, work atmosphere or working condition, promotion or career growth and partner or team cohesion. In addition to these factors, this study includes three more dimensions—training and development opportunities, organizational culture and policy, and job security.

In Bangladesh, an important factor related to employee motivation is the compensation package. Moreover, the management of private universities in Bangladesh usually does not pay much attention to academics’ compensation, career growth and facilities in the workplace, because most of the private universities are managed and sponsored by businessmen for whom profits are the ultimate aim. Job security is another important factor affecting job satisfaction in Bangladesh, because of the increasing unemployment rate. Moreover, there is no doubt that academic staff training and development programs can contribute to staff satisfaction and increase the morale of academics. However, training and development may not affect academics’ turnover decisions significantly. Healthy working conditions motivate employee retention. Sometimes, such conditions reduce employee turnover and increase job satisfaction among employees. Employees may be interested in joining a company for its well-established culture and policies. Some studies showed a positive link between job satisfaction and strong organizational culture regarding employee retention practices. Previous studies reported that the relationship with the supervisor is more emphasized in Asian countries. Thus, it is highly expected in the Asian context that a good relationship between subordinates and the boss be given priority. As a team member, an employee is more likely to stay in an organization for the strong teamwork affiliation they have created in the workplace. Thus, team cohesion is another important factor related to job satisfaction for private university academics in Bangladesh.

## Methodology

### Sample and data collection procedure

This study is conducted among academic staffs, appointed as full-time Lecturers, Assistant Professors, Associate Professors and Professors from ten private universities (among 74 private universities) in Bangladesh namely American International University Bangladesh, BRAC University, Daffodil International University, East West University, International Islamic University Chittagong (Dhaka Campus), Independent University Bangladesh, North South University, Southeast University, University of Liberal Arts Bangladesh, and United International University. The universities are located in Dhaka, the capital city of Bangladesh. Here, private universities are selected instead of public universities as HR practices and policies are the same in all public universities whilst it varies in private universities.

For selecting sample size, some factors such as population size, precision and confidence, time and cost constraints were considered. Four hundred (400) questionnaires were sent out based on a non-probability sampling technique. The response rate was 86.5% with 346 useable questionnaires. The respondents were from different faculties and a mixture between business and non-business academics. In this study, personal interviews were further conducted to acquire the necessary information from the respondents.

### Measurement instrument

In this study, a closed-ended questionnaire on job satisfaction was developed for academics of private universities in Bangladesh using a Likert scale (where1 = strongly disagree and 7 = strongly agree). The questionnaire was developed based on literature of job satisfaction to suit the local context. Here, sixty four questions including 60 items/variacles were included on job satisfaction following the observations of Gibson, Gibson and Ingold [8] and Warr, Cook and Wall [57]. The questionnaire was split into two segments: demography and job satisfaction.

### Statistical tools for data analysis

This study consists of two analytical steps. Step 1 compiles the demographic data of the 346 respondents which includes gender, age, marital status, rank, education and monthly income. Step 2 consists of exploratory factor analysis (EFA) to determine the most significant factors for academics’ job satisfaction. In addition to these two steps, Pearson product moment correlations were tested among the extracted factors to determine which of them have a high correlation with the dependent variable—academics’ job satisfaction. Finally, a multiple regression analysis was run to examine which of the extracted factors have the strongest impact on academics’ job satisfaction in Bangladesh. These analyses were computed using the software SPSS version 20.0.

### Ethical considerations

Ethical approval was provided by the International Islamic University Chittagong (IIUC) ethics committee (reference: IIUCEC/05/Q0706/01/2014), and the study was performed in accordance with the national ethics regulations. The participants were informed of the purpose of the study and assured of confidentiality, and in turn, they provided their written consent.

## Results

The demographic results show that 61.56% (n = 213) of the participants are male academics, while 38.44% (n = 133) are female academics. In relation to position, 63.79% (n = 139) of the respondents identify as “Lecturer” 47.11% (n = 163), 27.16% (n = 94) as “Assistant Professor”, 17.92% (n = 62) as “Associate Professor”, and 7.81% (n = 27) as “Professor”. In terms of education, 63.87% (n = 221) respondents are master’s degree holders, 24.28% (n = 84) have bachelor’s degrees and 11.85% (n = 41) have MPhil or PhD degrees. The maximum basic monthly salary of USD1500 is earned by 10.12% of the respondents followed by those earning USD1201-USD1500 (17.05%), whilst 84 (24.28%) of the respondents have an income range of USD801-USD1200 and 168 (48.55%) earn between USD 500-USD 800.

Cronbach’s coefficient (α) for each dimension of the survey ranged from 0.72 to 0.91 (typically 0.6 and above is acceptable), which indicates the internal consistency of each dimension (factor) of job satisfaction [58, 59]. The Central Limit Theorem is applied for this large sample (346 academics), and therefore, there is no issue surrounding the normality of the data. In this relationship, MacCallum, Widaman, Zhang and Hong [60] stated that if all communalities of a sample are above 0.5, the sample is perfectly adequate. The extracted communalities (h^2^) of the variables were found to be between 0.531 and 0.799. Therefore, the sample size (346) of this study was suitable for factor analysis. The KMO value for the overall matrix was 0.821, which indicates that the sample size was statistically significant for factor analysis. Bartlett’s test of sphericity [61] was applied to verify the appropriateness of the data for factor analysis. This test was also found to be significant, having a value of less than 0.5.

In factor analysis, principal component analysis (PCA) followed by the varimax rotation was employed to identify the most influential factors of job satisfaction. There were 60 items in the study labelled X1, X2,‥‥, X60. Table 1 shows item loading values, Cronbach’s alpha and Eigen values. The variable loading value of above 0.50 are considered significant variable [62]. Some variables (i.e., X3, X5, X11, and X12) were dropped from the analysis due to low loading values. The60 items of job satisfaction scale were reduced to 33 items after exploratory factor analysis (EFA) was run. Six factors below extracted in the EFA together explained 68.53% of the total variance.

**Table 1:** Summary of Rotated Component Matrix, Cronbach’s alpha, variance and Eigen values.

Factor Name	Items	Item loading	Communalities (h^2^)	% of variance (Cumulative)	Cronbach’s Reliability Coefficient	Eigen value
Compensation package	X1	0.781	0.784	15.165 (15.165)	0.87	9.849
	X4	0.738	0.757			
	X6	0.709	0.623			
	X2	0.696	0.610			
	X7	0.663	0.602			
	X10	0.668	0.598			
	X8	0.581	0.586			
	X9	0.567	0.579			
Promotion	X15	0.795	0.770	15.039 (30.204)	0.72	4.675
	X14	0.781	0.789			
	X18	0.765	0.769			
	X16	0.678	0.678			
	X13	0.543	0.542			
	X20	0.512	0.516			
Job Security	X29	0.790	0.787	14.316 (44.521)	0.89	3.123
	X24	0.745	0.741			
	X28	0.689	0.679			
	X26	0.612	0.601			
	X25	0.599	0.567			
	X22	0.491	0.539			
Working Conditions	X35	0.765	0.799	10.732 (55.253)	0.91	2.078
	X32	0.679	0.667			
	X38	0.579	0.574			
	X34	0.550	0.565			
	X36	0.521	0.560			
Team Cohesion	X43	0.734	0.745	7.267 (62.520)	0.78	1.298
	X42	0.721	0.721			
	X45	0.634	0.612			
	X47	0.519	0.568			
Supervisory Support	X53	0.789	0.788	5.680 (68.200)	0.84	1.091
	X60	0.678	0.689			
	X58	0.560	0.589			
	X54	0.498	0.531			

**Source:** Developed by the authors on the basis of data collected for the present study.

- The extracted first factor is **compensation package**. This factor is represented by eight variables (X1, X4, X6, X2, X7, X10, X8 and X9) of job satisfaction, which accounted for 15.165% of the variance. The factor loadings of the variables range from 0.567 to 0.781. The eight items are: teachers’ pay is equivalent to that of others in similar positions at other universities; satisfactory rules exist regarding salary increases; satisfactory payment is received for extra work; satisfactory benefits packages are received by the employee; poor vacation & leave allowances are offered; insufficient rewards exist for those who work here; and good welfare facilities are provided.
- The extracted second factor is **career growth**. This factor is represented by six variables (X15, X14, X18, X16, X13, and X20) of job satisfaction, which accounted for 15.039% of the variance. The factor loadings of the variables range from 0.512 to 0.795. The six items are: good possibilities for promotion when one fulfills his or her promotion requirements; equal probability to be promoted to a superior position; transparent performance evaluation policies; dissatisfactory rules and policies for promotion; no recognition of Chairman’s effort; and Chairman’s satisfaction with his or her possibilities for promotion.
- The extracted third factor is **job security**. This factor is represented by six variables (X29, X24, X28, X26, X25, and X22) of job satisfaction, which accounted for 14.316% of the variance. The factor loadings of the variables range from 0.491 to 0.790. The items are: satisfaction with present job in terms of job security; job turnover rate is very high; rate of disciplinary action is high; permanent job is more important; improvement period for feedback result given by students or chairman is insufficient; and evaluation of teaching efficiency by student is acceptable. The latter item is included because of its strong relationship with this factor, though it has low loading on this factor.
- The extracted fourth factor is **working conditions**. This factor is represented by five variables (X35, X32, X38, X34, and X36) of job satisfaction, which accounted for 10.732% of the variance. The factor loadings of the variables range from 0.521 to 0.765. The items are: maintains clean and healthy working conditions; allows casual dress in the work place; flexible duty and responsibility roster; treats academics with dignity and respect; and encourages ethical behavior.
- The fifth extracted factor is **team cohesion**. This factor is represented by four variables (X43, X42, X45, and X47) of job satisfaction, which accounted for 7.267% of the variance. The factor loadings of the variables range from 0.519 to 0.734. The items are: working relationships of teachers are poor; mutual communication among the academics is good; dissimilar views are valued; and no one dictates over others.
- The sixth extracted factor is **supervisory support**. This factor is represented by four variables (X53, X60, X58, and X54) of job satisfaction, which accounted for 5.68% of the variance. The factor loadings of the variables range from 0.498 to 0.789. The items are: Chairman of the department or Dean of the faculty is cooperative; Chairman of the department appreciates academics’ suggestions; Chairman of the department provides advice for work improvement; and tasks and responsibilities are allocated unfairly. The last item is included because of its strong association with this factor, though it has low loading value.

Training & development as well as organizational culture & policy were dropped by PCA as the factor loading for the related variables were below 0.5.

Table 2 presents the mean, standard deviation as well as the Pearson product moment correlation between six factors and academics’ job satisfaction. The mean values for the six factors were found to be in a range of 4.746–6.077. The highest mean value was found for supervisory support and the lowest value was scored for career growth. In case of standard deviation values, the highest degree of deviation was scored for career growth and the lowest score was accounted for team cohesion with a value of 1.367 and 0.836 respectively. Table 2 reveals a significant positive correlation between academics’ job satisfaction and compensation package (r = .675, p<.01), followed by job security (r = .630, p<.05), working conditions (r = .575, p<.01), career growth (r = .521, p<.05), and teamwork cohesion (r = .435, p<.05). Supervisory support is not significantly correlated with job satisfaction.

**Table 2:** Mean, Standard Deviation and Correlation Coefficient.

Variables	Mean	Std. Dev.	SS	CP	WC	CG	TC	AJS
Supervisory Support (SS)	6.077	0.861						
Compensation Package (CP)	5.293	0.854	.266					
Working Conditions (WC)	5.673	0.846	.313*	.450**				
Career Growth (CG)	4.746	1.367	.161	.631**	.519**			
Team Cohesion (TC)	5.578	0.836	.251	.394**	.533*	.518**		
job security(JS)	4.791	0.984	.037	.656**	.665**	.752**	.545**	
Academics’ Job Satisfaction(AJS)	5.712	0.921	.228	.675**	.575**	.521*	.435*	.630*

Note: **p<.01, *P<.05, N = 346

Table 3 presents the results of the multiple regressions with six independent factors regressed against academics’ job satisfaction. The results show that compensation package (p<.001), job security (p<.01), supervisory support (p<.05) and working conditions (p<.05) are statistically significant influential factors of job satisfaction. The regression model is also significant at .000 level (F = 24.98, P<.001), and these six dimensions explain 31.4% of the total variation in academics’ job satisfaction in private universities of Bangladesh. Here, compensation package has the highest positive value of standardized coefficient (β), followed by job security, supervisory support, working conditions, career growth, and team cohesion.

**Table 3:** Results of Multiple Regression Analysis.

Variables	R2	Adjusted R2	F value	Std. β value	t-value
**Control variables**	**0.314**	**0.299**	27.63***
Compensation Package				0.513	4.471***
Job Security				0.397	3.589**
Supervisory Support				0.341	2.521*
Working Conditions				0.282	1.148*
Team Cohesion				0.013	1.001
Career Growth				0.126	0.162

Note: *p<.05, **p<.01, ***p<.001

## Discussions

A fairly high level of job satisfaction is observed among the university academicians. The results of this study reveal that the most important factor of job satisfaction for academics in private universities of Bangladesh is the compensation package. This finding is consistent with previous studies [17, 18]. Pay package is considered to be the most significant motivational factor in HR practice. For instance, the study on Nigeria conducted by Ovadje [16] found that the compensation package is a highly important variable influencing job satisfaction. Similarly, this factor was a great motivation tool in Pakistan to retain experienced academics [63]. However, the findings completely reject the outcomes of Khatri, Fern and Budhwar [22] who found that compensation package is not a significant factor in the job satisfaction research in Asian context. The previous result of Eisenberger, Stinglhamber, Vandenberghe, Sucharski and Rhoades [23] is also partially rejected, because the compensation package is scored as a significant job satisfaction factor for both junior and senior academics in the present study.

Bangladesh is suffering from an unemployment problem, with a current rate of unemployment of 38%. Consequently, employees always seek job security, and academics are no exception. Researchers opined that, around the world, the highest priority of academics is job security with regard to the issue of employee retention. Our results support previous research [3, 33, 34, 39] which concluded that job security increases job satisfaction. However, Chughtai and Zafar [38] argued that job satisfaction is slightly affected by job security is negated here.

Lastly, working conditions is observed as another significant factor related to academics’ job satisfaction. The results support the findings of other researchers [1, 31, 48] who stated that healthy working conditions might increase job satisfaction by reducing job stress among employees. The results of the present study contradict the assertion by Herzberg [51] that employees’ commitment may not be increased with better working conditions.

The results from Pearson product moment correlation indicate that stated six factors have positive influence on academics’ job satisfaction. Supervisory support has the score with the highest mean value. This indicates that academics are found to be highly satisfied with supervisory support among the universities. Interestingly, all the other factors of job satisfaction scored similar positive mean values. According to the respondents, training and development initiatives by university management constitute the least satisfactory factor. It is also worth mentioning here that the data range is relatively high in value among the variable of career growth. These results from standard deviation signify that universities are successfully maintaining the academics across the campuses.

In the multiple regression analysis, the dependent variable is academics’ job satisfaction (AJS) and six extracted factors are the independent variables. Compensation package, working conditions, job security, and supervisory support were identified having the strongest impact on the academics’ job satisfaction. This result is consistent with the result of factor analysis. The remaining two variables of the study namely team cohesion and career growth were not found statistically significant. Interestingly, supervisory support was not found significant in the correlation analysis while was found significant in the multivariate regression analysis due to the interaction with other variables.

### Implications of the study

There is little research regarding academics’ job satisfaction conducted in the context of developing countries, though there are mentionable research works in the context of western and developed countries. The findings of the present study will act as a bridge, filling a gap in the job satisfaction literature for countries with developing economies and, in particular, for Bangladesh. In Bangladesh, many private universities are facing severe turnover of skilled academics as dissatisfaction factors influence them to leave the organizations. To retain these experienced academics, the findings of the present study provide a guideline for university management to develop a strategic plan for exploring the job satisfaction factors with the most potential for academics of private universities in Bangladesh.

### Conclusions and recommendations

In this study, six factors (compensation package, career growth, job security, working conditions, supervisory support, and team cohesion) of academics’ job satisfaction among private universities in Bangladesh are extracted using exploratory factor analysis (EFA). These factors affect academics’ job satisfaction and influence their decision to either stay in or leave their job. Again, the job satisfaction factors are examined using several analytical methodologies i.e. correlation and regression analysis to identify the most influential factors for academics from the identified factors of job satisfaction by EFA. The three most influential factors for job satisfaction are compensation package, job security, and working condition. Interestingly, despite many other dissatisfaction issues of different degrees, respondents showed very positive attitudes toward the sense of pride in their job. Such an attitude truly reflects the optimism of university academics that they still consider teaching is a noble profession. Compared to many other job types, university academics are still not well paid. Based on the findings, it is recommended that the management of all private universities should take necessary steps to provide greater financial benefits and create supportive organizational culture. University management can incentivize employees for publications in top journals, give bonus and other benefits, etc. To boost job security, leaders need to show consideration for the morale, welfare and well-being of their team, and the organization must provide training to improve employee skills. Giving recognition is another strategy i.e. rewards for good teaching and research, fair and transparent policies for performance appraisal. Furthermore, university management should design HR strategies including management support, better infrastructure, flexible working conditions, team cohesion, and flexible rules and policies in a way that their academic staffs can also enjoy the maximum advantage of these strategies.

### Limitations and future research

This study has some limitations. *Firstly*, only eight factors of job satisfaction are examined for university academics in this study. Thus, more job satisfaction factors could be included in future studies. *Secondly*, this study can be extended to include the academics of public universities. *Thirdly*, the present study is conducted in only ten private universities situated in Dhaka. As a result, it may be difficult to generalize the findings of this study. For future research, a larger number of private universities and other establishments should be included in and out of Dhaka to better represent the private sector of Bangladesh.

## Supporting Information

S1 Dataset Supporting information. (PDF)
