# Supplementary material for: Determinants of Academics' Job Satisfaction: Empirical Evidence from Private Universities in Bangladesh
Source: PLoS One. 2015 Feb 20;10(2):e0117834. doi: 10.1371/journal.pone.0117834 (PMC4336319; doi:10.1371/journal.pone.0117834)
Supplement: S1 Dataset — (PDF) [file pone.0117834.s001.pdf]

Supporting Information

Demographic results

Particular	# respondent	%
Gender		
Male	213	61.56%
Female	133	38.44%
Designation		
Lecturer	163	47.11%
Assistant Professor	94	27.16%
Associate Professor	62	17.92%
Professor	27	7.81%
Academic qualification		
Bachelor’s degree	84	24.28%
Master’s degree	221	63.87%
MPhil or PhD degrees	41	11.85%
Monthly Income		
USD 500-USD 800	168	48.55%
USD801-USD1200	84	24.28%
USD1201-USD1500	59	17.05%
USD1501 and higher	35	10.12%

Supporting Information

Response to questionnaire (1-10)

	X1	X2	X3	X4	X5	X6	X7	X8	X9	X10
Respondent 1	7	6	4	7	4	6	7	6	7	3
Respondent 2	7	5	5	6	5	7	6	7	7	6
Respondent 3	6	7	7	7	7	6	7	7	6	5
Respondent 4	7	5	6	5	5	7	6	5	7	5
Respondent 5	7	4	6	7	4	7	7	7	7	6
Respondent 6	6	7	6	5	3	3	6	5	5	2
Respondent 7	4	6	5	7	2	6	7	2	7	5
Respondent 8	7	5	7	7	5	7	6	7	5	2
Respondent 9	5	5	2	6	7	5	5	7	7	2
Respondent 10	6	5	5	7	4	7	5	6	6	3
Respondent 11	7	4	6	5	3	6	6	7	7	5
Respondent 12	7	7	3	7	2	7	5	6	7	5
Respondent 13	4	7	6	6	5	4	6	7	6	6
Respondent 14	6	7	5	7	4	7	5	6	5	1
Respondent 15	5	6	5	6	7	6	7	7	7	6
Respondent 16	5	6	7	7	5	7	6	5	5	1
Respondent 17	7	5	3	6	4	4	7	7	6	6
Respondent 18	7	4	3	6	5	7	6	7	5	5
Respondent 19	7	3	6	6	5	6	4	6	7	7
Respondent 20	6	6	7	5	5	7	6	7	6	6
Respondent 21	5	7	5	7	6	3	5	5	7	4
Respondent 22	7	7	6	6	5	7	6	6	7	1
Respondent 23	5	4	6	7	7	6	4	6	7	3
Respondent 24	5	6	5	4	5	7	6	2	5	5
Respondent 25	6	4	2	5	5	5	3	4	7	6
Respondent 26	7	6	2	6	6	7	6	5	5	5
Respondent 27	7	6	6	6	5	6	4	7	7	6
Respondent 28	7	7	7	5	5	7	6	7	7	1
Respondent 29	6	6	6	7	7	7	7	7	7	5
Respondent 30	6	5	6	6	5	4	6	7	7	1
Respondent 31	6	7	6	5	6	7	7	6	6	1
Respondent 32	6	7	6	6	5	5	6	4	7	4
Respondent 33	5	7	6	7	5	7	6	7	7	3
Respondent 34	5	6	7	6	5	6	7	3	5	3
Respondent 35	5	6	5	5	7	7	6	5	7	3
Respondent 36	7	4	5	7	5	5	7	7	7	3
Respondent 37	7	6	7	7	5	7	6	7	7	4
Respondent 38	7	6	6	6	4	7	6	5	7	5
Respondent 39	6	6	6	6	3	7	7	7	6	6
Respondent 40	7	6	5	5	5	6	3	6	6	6
Respondent 41	7	7	3	5	4	7	5	7	7	1

Respondent 42	5	7	6	4	5	7	6	6	7	2
Respondent 43	7	7	2	4	3	7	5	4	7	2
Respondent 44	5	6	3	5	5	5	7	7	7	1
Respondent 45	7	6	6	6	4	7	3	7	7	2
Respondent 46	5	6	5	5	5	7	6	6	5	3
Respondent 47	4	6	2	4	5	6	4	7	7	1
Respondent 48	7	5	3	4	4	7	6	7	7	4
Respondent 49	6	6	6	4	5	7	7	7	7	6
Respondent 50	7	7	2	6	6	4	6	5	7	6
Respondent 51	5	7	6	5	5	7	6	7	6	1
Respondent 52	7	7	4	5	7	7	5	5	7	6
Respondent 53	7	6	6	4	5	5	6	5	6	6
Respondent 54	7	5	6	6	7	7	7	6	6	3
Respondent 55	7	6	4	7	5	7	6	7	5	5
Respondent 56	5	4	4	6	5	6	5	6	4	2
Respondent 57	7	6	6	5	4	4	6	5	7	1
Respondent 58	5	7	6	7	5	7	5	6	5	1
Respondent 59	6	6	6	7	5	6	6	7	7	6
Respondent 60	7	3	6	6	5	7	5	7	6	3
Respondent 61	7	6	6	4	6	7	6	6	6	2
Respondent 62	6	6	2	3	3	5	7	5	7	5
Respondent 63	7	4	2	7	5	7	6	5	7	3
Respondent 64	6	6	6	5	6	7	7	6	5	3
Respondent 65	6	6	2	3	5	5	6	5	6	6
Respondent 66	7	7	5	6	5	7	4	7	4	3
Respondent 67	6	6	2	7	6	7	6	6	4	5
Respondent 68	5	6	6	4	5	4	7	7	5	3
Respondent 69	7	7	6	7	5	7	7	7	7	6
Respondent 70	7	6	6	5	5	7	6	7	5	5
Respondent 71	5	6	6	7	6	5	6	6	6	4
Respondent 72	6	4	5	6	4	7	7	7	5	6
Respondent 73	4	6	2	7	5	6	6	5	5	1
Respondent 74	7	7	2	5	3	7	7	7	7	6
Respondent 75	7	6	6	7	5	6	5	7	6	6
Respondent 76	5	7	2	4	5	7	3	6	5	2
Respondent 77	6	6	2	5	5	5	5	7	5	2
Respondent 78	6	6	6	7	5	7	6	6	6	3
Respondent 79	7	5	6	6	4	7	5	6	7	6
Respondent 80	7	7	2	5	5	6	6	5	6	4
Respondent 81	3	3	2	6	7	7	3	5	6	6
Respondent 82	4	6	6	6	5	7	7	6	6	6
Respondent 83	5	6	6	5	5	5	6	6	7	1
Respondent 84	4	5	6	7	7	7	5	7	7	2
Respondent 85	5	7	6	5	5	6	5	8	7	2
Respondent 86	7	7	6	7	7	7	7	7	6	2
Respondent 87	7	5	7	6	5	7	5	6	5	3

Respondent 88	7	6	6	7	5	5	5	7	6	6
Respondent 89	7	6	2	5	7	7	7	7	6	3
Respondent 90	6	7	6	6	5	7	7	6	7	6
Respondent 91	7	6	3	7	5	6	4	6	7	3
Respondent 92	5	5	6	6	7	7	7	7	7	2
Respondent 93	6	5	3	7	5	7	5	6	6	6
Respondent 94	7	6	6	5	6	5	6	7	7	6
Respondent 95	3	6	6	7	5	7	7	6	6	2
Respondent 96	7	5	2	6	6	6	6	7	7	6
Respondent 97	2	6	4	5	7	5	7	5	5	3
Respondent 98	2	5	2	7	6	6	6	6	4	5
Respondent 99	4	5	6	4	7	7	7	7	4	2
Respondent 100	6	7	2	7	5	5	6	7	5	6
Respondent 101	7	6	3	6	5	5	7	6	7	3
Respondent 102	7	5	6	4	5	5	7	7	7	6
Respondent 103	5	7	3	5	7	5	6	6	6	4
Respondent 104	7	6	6	7	4	6	7	7	3	6
Respondent 105	6	5	6	6	5	7	7	6	6	3
Respondent 106	7	5	2	7	4	7	5	7	7	6
Respondent 107	7	7	6	2	5	6	6	4	5	2
Respondent 108	6	5	2	6	4	7	7	5	2	5
Respondent 109	7	6	6	7	4	5	5	7	5	2
Respondent 110	7	6	2	2	7	7	6	6	5	3
Respondent 111	5	5	6	7	3	6	6	6	5	6
Respondent 112	4	5	2	6	4	4	3	2	4	2
Respondent 113	7	6	6	7	4	5	6	5	7	6
Respondent 114	7	5	5	2	5	6	5	7	6	2
Respondent 115	7	7	5	5	5	7	6	7	7	2
Respondent 116	5	6	6	5	6	5	6	2	7	1
Respondent 117	7	7	6	6	5	6	7	2	5	6
Respondent 118	6	5	6	3	7	7	5	7	7	1
Respondent 119	7	6	6	6	5	5	6	7	7	6
Respondent 120	7	7	6	7	6	6	6	4	3	2
Respondent 121	6	5	6	6	5	7	5	7	6	6
Respondent 122	7	7	6	3	4	7	6	7	7	2
Respondent 123	7	6	2	7	5	5	6	7	7	6
Respondent 124	5	6	6	5	5	7	4	4	4	2
Respondent 125	7	7	5	4	5	7	6	5	5	2
Respondent 126	7	6	2	4	6	6	5	7	7	2
Respondent 127	4	7	4	7	5	7	6	7	7	7
Respondent 128	7	6	2	4	7	5	7	6	6	3
Respondent 129	7	6	2	7	5	6	7	7	5	7
Respondent 130	3	5	6	4	5	7	6	7	6	2
Respondent 131	7	6	4	3	5	7	5	7	7	3
Respondent 132	7	7	6	5	6	5	6	7	7	7
Respondent 133	5	6	2	6	5	7	6	5	6	5

Respondent 134	7	7	6	7	4	5	5	6	6	4
Respondent 135	7	6	5	6	5	5	7	5	6	6
Respondent 136	7	7	6	3	7	5	5	7	7	3
Respondent 137	6	7	6	4	5	5	6	5	5	1
Respondent 138	7	6	2	5	4	6	7	6	7	4
Respondent 139	7	7	2	2	5	7	6	6	7	7
Respondent 140	5	6	6	7	5	5	7	7	6	3
Respondent 141	7	7	3	7	5	6	6	7	7	2
Respondent 142	5	6	3	6	4	5	5	7	6	1
Respondent 143	7	6	5	7	5	6	7	6	6	5
Respondent 144	6	7	7	3	4	5	5	7	7	2
Respondent 145	7	6	3	7	5	6	6	6	6	6
Respondent 146	5	6	3	6	4	5	7	6	7	3
Respondent 147	7	7	7	7	5	6	6	7	6	6
Respondent 148	7	6	3	2	4	5	7	7	6	6
Respondent 149	7	7	3	6	4	7	7	7	6	3
Respondent 150	5	7	2	7	3	6	6	6	6	5
Respondent 151	7	5	2	6	5	5	7	7	6	6
Respondent 152	5	7	3	7	4	7	6	7	7	3
Respondent 153	7	6	7	3	5	6	6	6	6	6
Respondent 154	5	7	7	7	6	7	5	7	7	6
Respondent 155	5	7	3	5	5	5	6	7	6	6
Respondent 156	7	5	3	7	3	5	5	7	7	3
Respondent 157	7	7	2	4	5	5	5	5	7	6
Respondent 158	6	6	3	7	5	6	6	7	6	6
Respondent 159	7	6	6	4	6	7	5	5	6	6
Respondent 160	5	6	6	7	5	5	6	7	7	3
Respondent 161	7	6	2	4	7	6	5	7	7	6
Respondent 162	6	7	3	4	6	5	5	5	7	6
Respondent 163	7	6	3	5	5	6	6	7	6	4
Respondent 164	7	7	3	7	5	5	6	7	7	6
Respondent 165	5	6	5	5	5	6	6	5	7	6
Respondent 166	7	7	6	7	6	5	6	7	7	3
Respondent 167	6	6	6	5	5	7	7	5	7	1
Respondent 168	7	7	3	7	7	5	6	7	7	2
Respondent 169	7	7	3	5	5	7	7	5	6	6
Respondent 170	5	7	2	7	5	6	6	7	6	3
Respondent 171	7	6	3	5	5	7	6	7	7	3
Respondent 172	7	5	3	7	6	5	6	5	6	1
Respondent 173	7	6	3	5	5	7	6	4	7	2
Respondent 174	7	7	6	7	7	6	6	7	6	5
Respondent 175	6	6	4	5	5	7	4	6	6	1
Respondent 176	7	6	6	5	6	5	6	7	6	5
Respondent 177	7	5	3	7	5	6	4	6	6	6
Respondent 178	5	7	7	7	4	7	6	7	7	2
Respondent 179	7	7	6	6	5	5	4	7	7	3

Respondent 180	7	5	5	7	5	7	6	4	6	6
Respondent 181	5	7	3	4	5	7	3	6	6	2
Respondent 182	7	5	3	7	6	6	6	7	7	7
Respondent 183	5	6	2	5	5	7	6	5	6	6
Respondent 184	5	6	6	6	7	5	6	7	7	5
Respondent 185	5	7	6	7	5	7	6	4	7	1
Respondent 186	6	6	5	5	5	6	7	6	6	1
Respondent 187	7	6	1	7	5	7	6	7	7	2
Respondent 188	7	7	6	4	4	6	6	4	5	2
Respondent 189	6	6	2	7	5	7	6	5	5	5
Respondent 190	7	7	3	5	4	7	5	7	7	7
Respondent 191	6	6	7	7	4	6	4	7	7	2
Respondent 192	7	7	6	6	5	7	6	7	6	5
Respondent 193	7	6	3	7	4	7	7	7	7	7
Respondent 194	6	7	6	4	5	5	6	6	7	2
Respondent 195	7	6	2	7	4	7	7	6	6	5
Respondent 196	5	4	3	5	5	4	6	7	5	7
Respondent 197	7	7	6	7	3	7	7	6	6	3
Respondent 198	4	6	5	6	5	5	6	7	7	7
Respondent 199	7	6	3	7	4	7	6	7	7	3
Respondent 200	3	7	3	5	5	6	6	7	7	1
Respondent 201	7	6	2	7	6	4	7	4	5	3
Respondent 202	4	7	3	6	5	5	6	7	7	2
Respondent 203	7	7	3	7	7	7	7	4	6	6
Respondent 204	5	6	2	6	5	5	6	7	5	2
Respondent 205	6	3	2	7	7	7	7	7	7	6
Respondent 206	4	6	2	5	5	5	4	5	6	6
Respondent 207	7	7	3	7	5	4	6	7	6	2
Respondent 208	7	6	2	5	5	7	3	5	5	6
Respondent 209	5	5	2	6	5	5	6	7	6	2
Respondent 210	7	6	3	5	4	7	6	6	7	6
Respondent 211	4	6	7	7	5	6	6	5	6	6
Respondent 212	7	7	5	7	5	4	5	6	5	6
Respondent 213	5	6	3	5	4	7	6	7	7	2
Respondent 214	7	5	3	5	5	5	3	7	7	1
Respondent 215	4	6	6	7	4	3	6	5	7	2
Respondent 216	7	6	6	5	5	7	5	6	5	2
Respondent 217	7	5	3	7	5	5	6	7	7	1
Respondent 218	7	6	3	5	3	7	7	6	6	6
Respondent 219	6	7	7	6	7	5	6	5	7	1
Respondent 220	6	6	6	7	5	3	7	7	5	2
Respondent 221	6	5	3	6	3	5	6	7	7	1
Respondent 222	5	6	3	7	5	7	6	6	6	5
Respondent 223	7	7	3	6	3	5	5	7	7	1
Respondent 224	4	6	2	7	5	7	6	5	6	1
Respondent 225	5	6	6	6	5	5	5	7	5	2

Respondent 226	6	7	2	6	7	7	5	7	7	1
Respondent 227	7	6	2	6	5	3	6	4	5	3
Respondent 228	7	6	6	5	3	6	5	7	7	1
Respondent 229	4	4	2	6	5	7	7	7	5	1
Respondent 230	7	6	5	7	5	7	6	5	7	3
Respondent 231	5	7	3	5	5	3	7	7	7	1
Respondent 232	7	6	2	7	5	7	6	6	5	6
Respondent 233	6	6	3	5	7	7	7	4	5	2
Respondent 234	6	6	3	7	5	5	7	7	6	7
Respondent 235	7	5	2	5	5	7	6	7	7	2
Respondent 236	7	6	7	5	5	4	7	5	6	6
Respondent 237	7	7	2	7	5	7	6	5	7	2
Respondent 238	7	6	6	5	5	5	7	6	6	3
Respondent 239	7	7	3	6	5	5	7	7	7	7
Respondent 240	6	7	6	7	4	7	7	6	7	5
Respondent 241	7	6	7	5	5	5	7	7	7	3
Respondent 242	6	6	3	7	5	7	7	7	7	7
Respondent 243	6	5	2	5	5	6	6	7	7	3
Respondent 244	7	7	2	7	6	7	7	7	5	1
Respondent 245	7	6	5	5	5	5	6	7	7	1
Respondent 246	7	6	5	6	5	6	7	6	5	1
Respondent 247	6	7	3	7	7	7	6	7	7	2
Respondent 248	7	6	7	7	5	6	6	6	7	6
Respondent 249	5	4	6	5	6	7	5	5	6	3
Respondent 250	7	6	3	7	5	5	6	6	6	6
Respondent 251	6	6	6	6	5	7	4	7	7	3
Respondent 252	7	5	6	4	5	5	3	7	6	6
Respondent 253	7	7	3	7	5	7	6	7	7	2
Respondent 254	6	6	2	4	4	5	6	5	7	2
Respondent 255	7	7	5	2	5	4	5	6	6	2
Respondent 256	6	6	3	3	4	7	6	6	6	6
Respondent 257	7	6	5	7	3	7	5	4	7	2
Respondent 258	6	6	5	2	5	7	6	7	5	2
Respondent 259	6	5	3	7	5	7	5	7	7	2
Respondent 260	7	6	5	3	5	6	6	4	6	1
Respondent 261	6	6	3	7	5	7	6	7	7	3
Respondent 262	4	7	2	4	6	7	5	6	6	6
Respondent 263	3	6	6	7	4	6	7	5	6	1
Respondent 264	6	3	7	6	5	7	7	7	7	7
Respondent 265	7	6	2	7	5	4	6	5	6	1
Respondent 266	6	6	5	5	6	7	7	6	6	6
Respondent 267	7	4	3	7	5	7	6	7	7	2
Respondent 268	5	6	2	4	7	4	7	5	7	1
Respondent 269	7	6	3	7	5	7	4	7	7	1
Respondent 270	5	7	7	5	5	5	4	5	2	2
Respondent 271	7	6	2	7	4	7	4	7	7	1

Respondent 272	7	7	3	6	5	6	6	6	6	6
Respondent 273	7	6	6	7	3	7	6	7	7	7
Respondent 274	5	6	2	4	5	5	6	7	7	1
Respondent 275	7	6	3	7	4	5	6	7	7	2
Respondent 276	6	5	3	4	5	6	6	7	7	2
Respondent 277	7	6	3	7	5	5	7	7	6	6
Respondent 278	5	4	6	5	5	6	6	7	6	1
Respondent 279	7	6	2	7	5	7	7	6	7	3
Respondent 280	6	7	7	3	6	6	6	7	7	2
Respondent 281	7	6	2	7	5	7	6	7	6	3
Respondent 282	4	6	2	2	7	7	4	7	5	5
Respondent 283	7	3	2	2	5	5	6	6	6	2
Respondent 284	5	6	3	4	5	7	4	6	7	6
Respondent 285	7	5	2	7	6	5	6	7	6	1
Respondent 286	7	6	3	5	5	5	3	6	6	6
Respondent 287	6	6	5	7	4	5	5	7	7	2
Respondent 288	7	7	2	2	5	5	6	7	6	7
Respondent 289	5	6	7	7	3	5	3	6	6	2
Respondent 290	7	6	2	6	5	5	6	7	7	6
Respondent 291	5	6	6	2	5	7	4	7	7	1
Respondent 292	5	7	5	7	6	6	6	6	7	3
Respondent 293	7	6	6	4	5	7	3	5	7	6
Respondent 294	5	5	6	7	5	6	6	6	5	3
Respondent 295	6	6	6	3	7	5	6	7	7	3
Respondent 296	7	6	3	7	5	7	6	6	7	4
Respondent 297	6	4	5	5	5	5	6	7	7	2
Respondent 298	7	6	5	7	6	7	6	6	7	3
Respondent 299	4	5	7	5	4	5	7	7	7	7
Respondent 300	7	6	2	7	5	7	6	7	7	4
Respondent 301	5	6	5	4	3	4	7	6	6	5
Respondent 302	7	7	2	7	3	7	6	7	7	3
Respondent 303	3	6	7	7	5	7	6	6	7	6
Respondent 304	7	6	5	4	5	6	7	7	7	7
Respondent 305	4	6	7	7	5	7	6	6	7	3
Respondent 306	5	2	2	5	6	4	6	7	7	7
Respondent 307	5	6	7	7	5	7	7	6	6	3
Respondent 308	7	3	7	7	4	7	6	7	7	7
Respondent 309	6	6	5	4	5	3	5	5	6	3
Respondent 310	5	4	5	7	6	7	6	6	7	7
Respondent 311	7	6	2	7	5	7	6	7	7	6
Respondent 312	5	4	6	5	7	7	7	7	6	6
Respondent 313	5	6	5	7	5	7	6	5	7	3
Respondent 314	5	5	3	6	5	4	5	7	7	6
Respondent 315	7	6	6	7	5	7	6	7	6	5
Respondent 316	7	5	3	7	5	5	5	5	7	3
Respondent 317	7	6	7	7	5	7	6	7	7	7

Respondent 318	5	7	5	6	4	6	6	7	7	6
Respondent 319	7	5	5	7	4	7	6	5	7	3
Respondent 320	7	6	3	5	4	5	6	6	5	6
Respondent 321	5	5	2	2	5	7	6	7	6	3
Respondent 322	4	6	2	6	4	7	5	5	7	3
Respondent 323	6	6	6	7	5	5	6	7	6	2
Respondent 324	7	6	2	6	4	7	5	7	7	7
Respondent 325	6	5	3	7	5	5	6	5	7	3
Respondent 326	5	6	2	5	4	7	6	7	7	7
Respondent 327	4	7	2	2	6	6	5	6	6	6
Respondent 328	5	6	6	5	5	7	6	5	7	4
Respondent 329	6	7	4	7	6	7	7	7	7	7
Respondent 330	5	6	6	6	5	5	6	5	7	3
Respondent 331	5	7	5	7	4	7	6	6	6	5
Respondent 332	7	6	4	5	5	7	6	7	6	4
Respondent 333	5	7	7	7	5	6	6	5	5	3
Respondent 334	7	6	3	5	5	7	7	7	6	2
Respondent 335	7	5	5	7	5	5	6	5	7	2
Respondent 336	6	6	4	3	5	6	6	5	6	3
Respondent 337	5	7	5	7	4	7	7	4	7	4
Respondent 338	7	6	5	2	5	7	6	5	5	4
Respondent 339	5	7	6	7	5	5	6	4	4	5
Respondent 340	7	6	3	2	5	7	5	6	4	4
Respondent 341	6	7	3	7	3	6	6	7	7	3
Respondent 342	7	6	4	7	5	7	6	5	7	3
Respondent 343	5	5	5	5	5	6	5	3	6	4
Respondent 344	7	6	5	7	5	7	6	6	7	4
Respondent 345	6	4	6	6	5	5	7	5	7	2
Respondent 346	7	6	7	7	6	7	6	6	5	3

Response to questionnaire (11-20)

	X11	X12	X13	X14	X15	X16	X17	X18	X19	X20
Respondent 1	5	5	5	7	7	7	7	7	2	4
Respondent 2	7	7	7	7	7	7	6	7	1	7
Respondent 3	4	6	6	7	4	7	7	6	3	5
Respondent 4	7	6	7	6	7	6	6	7	1	4
Respondent 5	7	6	5	7	7	7	7	7	4	6
Respondent 6	7	7	7	7	7	7	7	5	3	7
Respondent 7	6	7	6	6	6	5	2	5	3	4
Respondent 8	7	7	7	7	7	7	7	7	2	6
Respondent 9	5	6	5	7	7	7	7	7	2	7
Respondent 10	5	6	7	4	7	7	7	6	1	7
Respondent 11	7	6	7	7	6	5	6	6	3	7
Respondent 12	5	7	5	5	6	5	7	5	4	5
Respondent 13	5	5	6	7	2	7	7	7	1	6
Respondent 14	7	7	7	5	6	7	7	6	2	6
Respondent 15	5	7	7	5	6	7	7	7	1	7
Respondent 16	4	5	6	7	6	6	5	5	2	7
Respondent 17	5	7	6	7	5	6	5	7	3	7
Respondent 18	4	7	6	7	7	7	7	7	2	7
Respondent 19	4	5	7	7	7	7	7	6	2	4
Respondent 20	7	7	7	6	6	6	4	7	1	7
Respondent 21	7	6	7	7	7	7	7	7	1	7
Respondent 22	5	5	6	7	7	7	7	7	4	5
Respondent 23	4	6	5	7	6	7	6	7	2	6
Respondent 24	6	4	5	7	7	6	7	6	2	5
Respondent 25	4	6	6	7	7	7	6	7	2	7
Respondent 26	6	5	5	7	6	7	7	7	2	5
Respondent 27	5	5	6	7	6	6	7	6	1	6
Respondent 28	5	6	7	7	7	7	7	7	2	5
Respondent 29	6	5	5	7	6	7	7	7	2	7
Respondent 30	5	7	7	7	7	6	7	7	1	7
Respondent 31	4	7	6	7	6	7	7	5	3	6
Respondent 32	5	5	7	5	7	5	6	4	3	6
Respondent 33	6	6	7	7	6	6	7	7	1	7
Respondent 34	4	4	7	7	5	5	2	6	4	7
Respondent 35	6	4	6	5	4	5	4	5	2	6
Respondent 36	6	5	6	7	7	7	7	7	3	7
Respondent 37	6	7	7	2	6	5	7	5	1	7
Respondent 38	6	7	7	6	5	5	7	5	1	7
Respondent 39	4	6	6	5	6	7	7	7	1	6
Respondent 40	2	5	5	5	7	7	5	5	1	5
Respondent 41	7	7	7	7	7	7	7	7	2	7
Respondent 42	5	7	7	7	7	7	7	7	1	7

Respondent 43	7	7	6	7	7	6	7	6	4	6
Respondent 44	7	7	7	6	7	7	7	7	1	7
Respondent 45	7	7	7	7	5	6	7	7	1	7
Respondent 46	6	6	6	7	6	6	7	7	2	7
Respondent 47	7	7	7	5	7	7	7	7	3	6
Respondent 48	4	7	7	7	7	6	4	4	3	7
Respondent 49	7	7	7	7	7	7	7	7	1	7
Respondent 50	7	6	6	6	6	7	6	4	1	7
Respondent 51	7	5	7	7	7	7	7	7	2	7
Respondent 52	6	6	6	7	7	7	7	7	1	7
Respondent 53	6	5	6	7	7	7	6	7	1	5
Respondent 54	6	5	6	6	7	6	7	7	2	6
Respondent 55	5	6	5	7	7	6	5	4	3	5
Respondent 56	7	5	5	7	6	7	7	7	2	6
Respondent 57	5	5	5	7	7	7	7	7	2	5
Respondent 58	6	5	5	6	5	6	7	6	3	4
Respondent 59	7	6	7	6	7	7	6	7	1	5
Respondent 60	7	6	7	6	5	7	6	7	1	6
Respondent 61	5	5	5	7	7	5	7	7	1	7
Respondent 62	6	5	5	6	7	7	4	5	3	5
Respondent 63	6	7	6	7	7	6	6	5	1	7
Respondent 64	6	6	6	5	7	6	7	7	1	6
Respondent 65	4	5	6	2	2	6	4	5	3	5
Respondent 66	4	7	2	7	4	4	4	7	4	4
Respondent 67	6	6	5	7	7	7	7	7	2	6
Respondent 68	6	6	6	7	7	7	7	7	2	7
Respondent 69	7	7	7	7	7	7	6	7	1	6
Respondent 70	6	6	5	6	7	7	5	7	2	7
Respondent 71	4	6	5	7	6	6	7	6	2	7
Respondent 72	4	7	6	7	7	7	5	6	4	7
Respondent 73	4	5	6	7	6	5	7	5	2	6
Respondent 74	7	6	5	6	7	7	4	6	2	6
Respondent 75	4	6	7	6	5	7	7	5	2	7
Respondent 76	5	6	5	6	7	6	6	5	4	4
Respondent 77	7	7	5	7	7	7	7	7	2	7
Respondent 78	5	6	5	5	7	6	7	5	2	6
Respondent 79	6	5	6	6	7	5	6	5	4	7
Respondent 80	2	6	5	6	5	5	5	7	1	7
Respondent 81	4	6	6	5	6	7	4	2	2	5
Respondent 82	5	7	7	6	5	6	6	6	2	6
Respondent 83	5	6	7	6	6	7	7	6	2	7
Respondent 84	7	6	7	6	7	7	4	6	2	7
Respondent 85	2	7	5	7	5	6	7	7	2	6
Respondent 86	6	6	7	4	7	6	7	6	1	7
Respondent 87	4	7	6	7	7	7	5	6	2	6
Respondent 88	6	6	7	7	6	7	4	4	3	7

Respondent 89	5	5	6	6	6	7	6	6	2	7
Respondent 90	7	7	6	7	7	7	7	7	2	6
Respondent 91	7	7	7	6	6	6	7	6	1	7
Respondent 92	4	6	7	6	6	7	7	7	2	7
Respondent 93	4	7	6	6	7	6	6	5	2	6
Respondent 94	7	6	7	6	7	6	7	7	2	7
Respondent 95	7	6	6	7	6	7	7	6	2	7
Respondent 96	7	7	7	5	7	6	7	7	1	7
Respondent 97	7	5	6	4	7	6	4	4	2	5
Respondent 98	5	6	5	6	7	6	6	5	1	6
Respondent 99	5	5	6	5	5	5	2	4	3	5
Respondent 100	5	7	5	6	7	6	7	4	3	6
Respondent 101	7	7	7	6	6	6	7	6	1	7
Respondent 102	4	6	7	6	7	7	7	7	2	7
Respondent 103	4	7	6	6	7	6	7	5	2	6
Respondent 104	7	6	7	6	7	6	7	7	2	7
Respondent 105	7	6	6	7	7	7	7	6	2	7
Respondent 106	7	5	6	5	7	6	7	7	1	7
Respondent 107	7	4	5	4	7	6	4	4	2	5
Respondent 108	5	6	5	6	5	6	6	5	1	6
Respondent 109	5	5	6	5	7	6	2	4	3	5
Respondent 110	5	7	5	6	7	6	7	4	3	6
Respondent 111	5	5	6	6	5	6	5	5	2	5
Respondent 112	7	6	5	7	6	7	6	7	2	6
Respondent 113	7	7	6	7	5	6	6	5	3	2
Respondent 114	5	6	7	7	7	6	7	7	1	7
Respondent 115	7	7	6	7	7	7	7	6	1	7
Respondent 116	7	6	6	5	5	5	6	4	2	6
Respondent 117	7	6	6	5	5	6	6	4	3	6
Respondent 118	7	6	6	7	7	6	7	7	1	6
Respondent 119	7	7	7	7	6	6	7	7	2	7
Respondent 120	6	5	7	5	7	5	2	4	1	6
Respondent 121	7	5	6	7	6	7	7	7	2	6
Respondent 122	7	7	7	7	7	7	5	7	1	7
Respondent 123	7	7	7	7	6	7	7	7	2	7
Respondent 124	4	6	5	7	4	5	4	4	3	6
Respondent 125	6	6	6	7	5	7	7	5	3	6
Respondent 126	7	6	7	7	7	7	7	7	1	7
Respondent 127	6	5	7	7	7	7	7	7	1	7
Respondent 128	6	7	5	7	6	7	7	6	4	7
Respondent 129	7	7	7	7	6	7	7	7	1	4
Respondent 130	6	6	6	7	7	6	7	7	2	7
Respondent 131	7	7	7	6	7	7	7	7	1	5
Respondent 132	7	7	6	7	6	6	7	7	1	6
Respondent 133	4	6	6	7	7	6	7	5	1	7
Respondent 134	6	7	6	7	6	7	7	6	1	7

Respondent 135	5	6	7	6	6	7	4	5	2	6
Respondent 136	5	7	6	7	7	7	7	7	1	7
Respondent 137	7	7	6	7	7	7	4	6	1	7
Respondent 138	6	7	6	7	7	6	7	6	1	7
Respondent 139	7	7	6	7	7	6	7	7	1	7
Respondent 140	7	7	6	7	7	6	6	7	1	6
Respondent 141	6	6	6	6	7	7	7	7	1	7
Respondent 142	7	7	6	6	6	6	6	6	1	7
Respondent 143	5	5	5	6	6	7	6	7	2	6
Respondent 144	6	6	6	5	6	7	7	7	2	6
Respondent 145	6	6	6	7	7	6	5	7	2	5
Respondent 146	6	6	6	6	5	6	6	7	2	6
Respondent 147	6	6	5	5	7	7	6	7	2	6
Respondent 148	5	5	5	6	6	7	6	7	1	7
Respondent 149	6	7	7	6	7	6	6	6	3	6
Respondent 150	5	6	6	6	6	7	7	7	3	5
Respondent 151	7	6	6	6	6	7	7	7	2	7
Respondent 152	7	7	7	5	6	6	7	7	1	7
Respondent 153	7	6	7	6	6	7	7	7	2	6
Respondent 154	7	6	6	6	7	7	7	7	2	6
Respondent 155	6	6	6	5	7	7	6	6	1	6
Respondent 156	6	6	6	7	7	7	6	7	2	6
Respondent 157	7	7	6	6	6	7	6	7	2	6
Respondent 158	7	7	6	7	7	7	7	7	2	7
Respondent 159	6	6	7	7	7	7	6	7	1	6
Respondent 160	6	6	7	7	6	6	7	7	2	5
Respondent 161	7	7	7	6	7	5	7	7	1	7
Respondent 162	6	5	7	5	7	7	7	7	2	7
Respondent 163	7	7	7	5	7	6	7	7	1	6
Respondent 164	5	5	6	4	7	7	7	7	2	6
Respondent 165	6	6	6	4	6	7	7	7	2	6
Respondent 166	6	6	7	7	7	7	7	7	1	7
Respondent 167	7	7	7	7	7	7	6	7	2	7
Respondent 168	6	6	6	6	6	5	6	7	2	6
Respondent 169	7	6	6	5	6	6	5	7	2	7
Respondent 170	6	6	7	5	7	7	6	7	1	7
Respondent 171	7	7	7	7	7	7	7	7	1	7
Respondent 172	6	6	6	6	6	6	6	6	2	6
Respondent 173	7	7	7	7	7	7	7	7	1	7
Respondent 174	4	5	5	5	6	6	6	6	2	6
Respondent 175	6	6	6	6	6	6	6	6	2	6
Respondent 176	4	7	6	6	6	6	6	6	3	6
Respondent 177	6	6	6	6	6	6	6	6	2	6
Respondent 178	7	7	7	7	7	7	7	7	1	7
Respondent 179	7	7	5	7	6	7	7	6	1	7
Respondent 180	6	6	6	6	6	6	6	6	2	6

Respondent 181	6	6	6	6	6	6	6	6	2	7
Respondent 182	7	7	7	7	7	7	7	7	1	7
Respondent 183	5	6	6	6	6	6	6	6	3	4
Respondent 184	2	6	6	6	5	6	6	6	3	4
Respondent 185	7	7	7	7	7	7	7	7	1	7
Respondent 186	6	6	6	6	6	6	6	6	2	6
Respondent 187	7	7	7	7	7	7	7	7	1	7
Respondent 188	4	5	5	5	5	6	5	6	2	2
Respondent 189	5	5	5	5	5	5	5	5	3	5
Respondent 190	7	7	7	7	7	7	7	7	2	6
Respondent 191	7	7	7	7	7	7	7	7	1	7
Respondent 192	4	6	6	5	5	5	5	5	3	2
Respondent 193	2	7	7	7	7	7	7	7	1	7
Respondent 194	4	7	7	7	6	7	6	7	1	7
Respondent 195	6	5	5	5	5	5	5	5	3	2
Respondent 196	7	7	7	7	7	7	7	7	1	7
Respondent 197	4	5	5	5	5	6	5	5	2	2
Respondent 198	7	7	7	7	7	7	7	7	1	7
Respondent 199	7	6	6	6	6	6	6	6	2	6
Respondent 200	7	7	7	7	7	7	7	7	1	7
Respondent 201	6	6	6	6	7	7	6	7	2	6
Respondent 202	7	6	6	7	6	7	7	7	2	6
Respondent 203	7	6	6	6	6	6	6	5	2	6
Respondent 204	6	5	6	6	7	7	7	7	3	5
Respondent 205	7	6	6	7	6	6	6	7	2	6
Respondent 206	6	6	6	6	6	7	6	7	2	6
Respondent 207	6	6	6	7	6	7	7	7	2	6
Respondent 208	6	6	6	6	6	4	7	7	3	6
Respondent 209	7	7	6	6	7	7	7	7	2	6
Respondent 210	7	7	7	7	7	7	6	6	1	7
Respondent 211	6	6	6	6	6	6	6	6	2	6
Respondent 212	6	6	6	6	6	6	6	6	2	6
Respondent 213	7	7	7	7	7	7	7	7	1	7
Respondent 214	7	7	7	7	7	7	7	7	1	7
Respondent 215	7	7	7	7	7	7	7	7	1	7
Respondent 216	4	6	6	6	5	6	6	6	3	4
Respondent 217	7	7	7	7	7	7	7	7	1	7
Respondent 218	6	6	6	6	6	6	6	6	2	6
Respondent 219	7	7	7	7	7	7	7	7	1	7
Respondent 220	7	7	7	7	7	7	7	7	1	7
Respondent 221	7	7	7	7	7	7	7	7	1	7
Respondent 222	7	6	6	6	6	6	6	6	2	6
Respondent 223	7	7	7	7	7	7	7	7	1	7
Respondent 224	4	6	6	6	6	6	6	6	3	5
Respondent 225	7	7	7	7	7	7	7	7	1	7
Respondent 226	7	7	7	7	7	7	7	7	1	7

Respondent 227	7	6	6	6	6	6	6	6	3	
Respondent 228	7	7	7	7	7	7	7	7	1	7
Respondent 229	7	7	7	7	7	7	7	7	1	7
Respondent 230	6	6	6	6	6	6	6	6	2	6
Respondent 231	7	7	7	7	7	6	7	7	1	7
Respondent 232	5	5	5	5	5	2	5	5	2	2
Respondent 233	4	6	6	6	7	6	6	7	2	7
Respondent 234	6	7	7	7	7	7	7	6	1	7
Respondent 235	6	7	7	7	7	7	7	7	1	7
Respondent 236	7	4	4	5	4	7	6	7	1	7
Respondent 237	5	5	5	5	5	6	6	5	3	2
Respondent 238	6	6	6	6	6	6	7	6	2	6
Respondent 239	7	7	7	7	7	6	6	6	1	7
Respondent 240	6	6	6	6	6	6	6	6	2	6
Respondent 241	7	7	7	7	6	7	7	7	1	7
Respondent 242	7	7	7	7	7	7	6	7	1	7
Respondent 243	7	4	6	6	6	7	7	7	1	7
Respondent 244	7	6	6	6	5	7	7	7	1	7
Respondent 245	6	6	6	6	7	6	5	7	2	7
Respondent 246	6	4	2	7	5	7	7	7	2	2
Respondent 247	7	7	7	7	7	7	7	7	1	7
Respondent 248	7	7	7	7	7	7	7	7	1	7
Respondent 249	7	6	6	7	7	7	7	7	2	6
Respondent 250	6	6	6	6	6	6	6	6	2	6
Respondent 251	5	5	5	5	5	6	7	7	2	5
Respondent 252	7	6	6	6	6	6	7	7	3	5
Respondent 253	7	7	7	7	7	7	7	7	1	7
Respondent 254	7	7	7	7	7	7	7	7	1	7
Respondent 255	6	6	6	6	6	6	6	6	2	6
Respondent 256	6	6	6	6	6	6	6	6	2	6
Respondent 257	7	7	7	7	7	7	7	7	1	7
Respondent 258	6	5	5	5	5	6	7	7	3	6
Respondent 259	7	7	7	7	7	7	7	7	1	7
Respondent 260	6	5	5	5	6	6	7	7	3	6
Respondent 261	7	7	7	7	7	7	7	7	1	7
Respondent 262	5	5	6	5	5	6	6	6	2	5
Respondent 263	5	5	7	5	6	6	6	6	2	2
Respondent 264	7	7	7	7	7	7	7	7	1	7
Respondent 265	6	6	6	6	6	6	6	6	2	5
Respondent 266	6	6	6	6	6	6	6	6	2	6
Respondent 267	6	6	6	6	6	6	6	6	2	6
Respondent 268	7	7	7	7	7	7	7	7	1	7
Respondent 269	7	7	7	7	7	7	7	7	1	7
Respondent 270	4	2	7	5	5	5	5	5	3	4
Respondent 271	7	7	7	7	7	7	7	7	1	7
Respondent 272	6	6	6	6	6	6	6	6	2	6

Respondent 273	6	6	6	6	6	6	6	6	2	6
Respondent 274	7	7	7	7	7	7	7	7	1	7
Respondent 275	7	7	7	7	7	7	7	7	1	7
Respondent 276	7	7	7	7	7	7	7	7	1	7
Respondent 277	5	5	5	5	5	5	6	7	2	6
Respondent 278	5	5	5	5	5	5	7	7	3	5
Respondent 279	6	6	7	6	6	6	6	6	2	6
Respondent 280	7	7	7	7	7	7	7	7	1	7
Respondent 281	7	7	7	7	7	7	7	7	1	7
Respondent 282	7	5	7	5	6	7	7	6	1	7
Respondent 283	7	6	5	6	7	7	7	7	1	7
Respondent 284	7	6	6	7	6	7	7	7	1	6
Respondent 285	6	6	6	7	7	7	6	6	1	6
Respondent 286	7	6	6	6	7	6	6	7	1	6
Respondent 287	6	6	7	6	6	6	7	7	1	6
Respondent 288	6	6	6	6	6	6	7	7	1	6
Respondent 289	7	7	6	6	7	6	6	7	2	6
Respondent 290	6	6	6	5	6	6	5	6	2	6
Respondent 291	7	5	7	7	7	6	6	5	2	7
Respondent 292	7	7	7	7	7	7	7	7	1	7
Respondent 293	6	7	7	7	6	7	6	5	3	6
Respondent 294	6	6	6	6	6	6	6	6	2	6
Respondent 295	7	6	7	7	7	7	7	7	1	7
Respondent 296	7	7	7	7	7	7	7	7	1	7
Respondent 297	7	7	7	7	6	7	7	7	1	7
Respondent 298	7	7	7	7	7	7	7	6	2	6
Respondent 299	7	7	5	7	7	7	7	7	2	7
Respondent 300	7	6	6	6	6	6	6	6	1	7
Respondent 301	6	6	6	7	7	7	5	7	1	7
Respondent 302	7	7	7	7	7	7	7	7	1	7
Respondent 303	7	7	7	7		6	7	7	2	6
Respondent 304	7	7	7	7	7	7	7	7	1	7
Respondent 305	7	7	7	7	7	7	5	6	1	7
Respondent 306	6	7	7	6	7	7	7	7	1	7
Respondent 307	7	7	7	7	7	7	7	7	2	7
Respondent 308	7	6	7	7	7	7	7	7	1	7
Respondent 309	4	4	5	6	4	5	5	7	1	7
Respondent 310	7	7	5	5	6	5	6	6	3	6
Respondent 311	7	6	6	7	7	7	7	7	2	7
Respondent 312	7	6	6	6	6	6	6	7	3	7
Respondent 313	7	7	5	7	6	7	6	6	3	6
Respondent 314	7	6	6	7	7	7	7	7	3	6
Respondent 315	7	6	6	7	7	7	7	7	3	7
Respondent 316	7	6	6	5	6	6	6	7	3	6
Respondent 317	7	6	6	7	7	7	7	6	2	7
Respondent 318	7	7	6	6	6	5	7	7	2	5

Respondent 319	7	6	6	6	6	7	7	7	2	7
Respondent 320	7	7	6	6	6	5	7	6	2	5
Respondent 321	6	7	7	7	6	7	6	7	1	7
Respondent 322	7	7	7	7	6	7	7	7	2	7
Respondent 323	7	6	6	6	7	7	7	7	1	6
Respondent 324	7	7	7	7	7	5	7	7	2	7
Respondent 325	6	7	7	7	7	7	6	7	1	6
Respondent 326	7	7	7	7	7	7	7	7	1	7
Respondent 327	7	6	6	7	6	5	7	6	2	6
Respondent 328	7	5	6	6	7	7	6	7	2	7
Respondent 329	7	7	7	7	7	7	7	7	1	6
Respondent 330	7	7	7	7	7	7	7	7	1	7
Respondent 331	6	6	6	6	7	6	5	6	2	7
Respondent 332	5	5	5	7	6	7	6	7	3	5
Respondent 333	7	7	7	6	7	7	6	5	3	5
Respondent 334	6	6	6	7	6	5	3	6	3	7
Respondent 335	6	5	6	6	6	6	4	6	4	7
Respondent 336	6	7	7	7	7	5	5	6	2	5
Respondent 337	5	6	6	6	7	6	6	5	3	7
Respondent 338	7	6	7	7	6	7	7	6	1	5
Respondent 339	5	7	6	6	7	6	6	7	1	6
Respondent 340	4	5	7	7	7	5	7	6	1	6
Respondent 341	6	6	7	6	6	5	6	4	3	5
Respondent 342	7	7	7	6	7	6	7	5	2	6
Respondent 343	6	6	6	7	7	7	7	4	2	7
Respondent 344	7	6	6	6	7	6	7	5	2	6
Respondent 345	6	7	6	5	6	7	5	6	1	7
Respondent 346	7	7	7	6	5	6	6	7	2	5

Response to questionnaire (21-30)

	X21	X22	X23	X24	X25	X26	X27	X28	X29	X30
Respondent 1	5	6	6	7	5	7	7	5	6	7
Respondent 2	7	5	7	7	5	7	7	5	7	7
Respondent 3	7	7	6	7	6	2	7	7	7	7
Respondent 4	5	5	6	7	5	7	7	2	7	7
Respondent 5	7	4	7	7	6	2	7	7	6	7
Respondent 6	7	7	6	6	5	5	4	5	7	7
Respondent 7	4	6	5	7	6	4	5	2	7	7
Respondent 8	2	5	4	7	6	7	7	2	7	7
Respondent 9	6	5	7	7	7	5	7	7	7	7
Respondent 10	4	5	7	5	4	7	2	7	7	5
Respondent 11	6	4	5	7	7	5	6	7	6	6
Respondent 12	6	7	7	7	7	5	7	7	5	7
Respondent 13	6	7	7	7	7	7	7	7	6	7
Respondent 14	2	7	7	5	7	7	7	6	6	7
Respondent 15	6	6	5	5	7	6	7	7	7	7
Respondent 16	5	6	7	7	5	5	5	5	5	5
Respondent 17	7	5	6	7	6	2	5	6	2	7
Respondent 18	2	4	7	7	7	6		6	7	7
Respondent 19	7	3	4	6	7	7	6	5	5	5
Respondent 20	7	6	6	7	7	7	7	7	2	7
Respondent 21	7	7	7	7	7	7	7	7	6	7
Respondent 22	6	7	7	7	6	7	7	7	7	7
Respondent 23	7	4	7	7	6	7	7	7	7	7
Respondent 24	6	6	6	7	6	5	7	6	7	7
Respondent 25	5	4	7	5	5	5	6	7	7	7
Respondent 26	5	6	5	6	6	5	7	5	6	7
Respondent 27	7	6	6	7	6	7	7	6	7	7
Respondent 28	7	7	6	7	7	7	7	7	5	7
Respondent 29	7	6	7	7	6	7	7	6	7	7
Respondent 30	7	5	7	7	7	7	7	7	7	7
Respondent 31	6	7	7	7	7	5	5	5	6	7
Respondent 32	7	7	6	7	7	4	6	4	7	4
Respondent 33	6	7	7	7	7	7	6	6	7	7
Respondent 34	2	6	4	7	7	2	5	2	5	7
Respondent 35	6	6	5	7	7	4	7	5	7	5
Respondent 36	7	4	7	7	7	6	5	7	7	4
Respondent 37	7	6	7	4	7	2	7	5	5	7
Respondent 38	5	6	5	7	6	6	7	2	5	6

Respondent 39	6	6	7	7	7	7	7	2	6	6
Respondent 40	6	6	5	7	6	7	7	6	4	7
Respondent 41	7	7	7	7	7	7	7	7	7	7
Respondent 42	7	7	7	7	7	4	7	7	5	7
Respondent 43	6	7	7	7	7	5	4	4	5	2
Respondent 44	7	6	7	7	7	7	7	7	7	7
Respondent 45	7	6	7	7	7	7	7	7	7	7
Respondent 46	6	6	7	7	7	6	7	5	5	5
Respondent 47	7	6	6	6	7	7	7	7	7	7
Respondent 48	7	5	6	5	6	7	7	5	7	7
Respondent 49	7	6	7	7	7	7	7	7	7	7
Respondent 50	7	7	7	4			7	6	7	7
Respondent 51	7	7	7	7	7	2	7	7	6	7
Respondent 52	7	7	7	5	5	6	7	5	6	7
Respondent 53	7	6	6	5	5	5	6	6	6	7
Respondent 54	6	5	5	5	5	6	6	6	7	6
Respondent 55	5	6	7	7	6	2	4	2	2	5
Respondent 56	7	4	5	5	5	5	2	7	6	7
Respondent 57	7	6	6	5	5	2	7	6	6	7
Respondent 58	6	7	7	7	7	2	6	5	5	7
Respondent 59	7	6	6	5	5	6	6	7	7	7
Respondent 60	7	3	6	7	6	5	7	7	6	2
Respondent 61	6	6	4	7	7	6	6	5	5	5
Respondent 62	6	6	7	7	7	5	6	7	7	7
Respondent 63	6	4	7	7	6	7	7	5	6	7
Respondent 64	6	6	6	5	5	6	6	5	6	6
Respondent 65	4	6	4	5	5	4	5	2	6	4
Respondent 66	4	7	4	4	4	7	2	5	6	6
Respondent 67	2	6	6	5	5	6	2	5	7	7
Respondent 68	6	6	7	7	6	6	6	6	6	6
Respondent 69	6	7	7	5	5	6	6	6	5	6
Respondent 70	5	6	5	7	7	5	6	2	6	7
Respondent 71	5	6	6	7	7	5	6	7	6	7
Respondent 72	5	4	5	7	7	4	7	6	6	7
Respondent 73	6	6	5	7	7	5	5	4	4	7
Respondent 74	5	7	6	7	7	6	5	5	6	7
Respondent 75	6	6	6	7	7	2	6	7	6	7
Respondent 76	2	7	5	6	7	5	7	6	6	6
Respondent 77	7	6	7	7	7	7	6	6	6	7
Respondent 78	6	6	6	7	7	5	6	6	5	7
Respondent 79	7	5	6	6	7	5	6	6	5	6
Respondent 80	5	7	5	7	7	2	6	5	5	7
Respondent 81	6	3	6	7	7	2	5	2	5	7
Respondent 82	6	6	7	7	7	6	6	5	6	7

Respondent 83	7	6	6	7	7	6	6	7	5	7
Respondent 84	6	5	6	7	6	5	6	7	7	7
Respondent 85	7	7	7	7	6	6	6	5	7	7
Respondent 86	6	7	6	7	7	6	6	7	6	7
Respondent 87	5	5	6	5	5	2	5	7	5	5
Respondent 88	7	6	7	7	6	4	2	5	6	7
Respondent 89	6	6	6	7	6	4	6	6	7	5
Respondent 90	6	7	7	7	6	7	7	7	6	5
Respondent 91	6	6	7	7	6	7	7	7	6	7
Respondent 92	5	5	6	7	7	4	6	7	6	7
Respondent 93	7	5	6	7	7	6	6	7	5	5
Respondent 94	7	6	6	7	7	7	7	7	6	7
Respondent 95	7	6	6	6	7	6	6	5	6	7
Respondent 96	7	5	6	7	7	6	7	7	6	7
Respondent 97	2	6	2	4	4	4	5	6	4	7
Respondent 98	7	5	5	7	7		2		5	4
Respondent 99	4	5	6	4	4	2	6	7	6	7
Respondent 100	5	7	7	6	6	7	6	7	5	6
Respondent 101	6	6	7	7	6	7	7	7	6	7
Respondent 102	2	5	6	7	7	4	6	7	6	7
Respondent 103	7	7	6	7	7	6	6	7	5	5
Respondent 104	7	6	6	7	7	7	7	7	6	7
Respondent 105	7	5	6	6	7	6	6	5	6	7
Respondent 106	7	5	6	7	7	6	7	7	6	7
Respondent 107	2	7	2	4	4	4	5	6	4	7
Respondent 108	7	5	6	7	7	6	2	5	5	4
Respondent 109	4	6	6	4	4	2	6	7	6	7
Respondent 110	5	6	7	6	6	7	6	7	5	6
Respondent 111	6	5	6	7	6	4	6	5	6	6
Respondent 112	7	5	6	6	6	7	6	7	6	6
Respondent 113	4	6	5	5	5	6	5	6	5	5
Respondent 114	6	5	6	7	6	7	6	7	6	6
Respondent 115	7	7	6	7	6	7	6	6	7	6
Respondent 116	7	6	7	7	7	6	7	6	7	6
Respondent 117	7	7	6	7	5	6	6	7	6	7
Respondent 118	7	5	6	7	6	6	6	7	7	6
Respondent 119	7	6	6	7	6	6	6	6	7	6
Respondent 120	6	7	6	6	6	7	6	6	5	6
Respondent 121	7	5	7	5	6	6	7	5	6	7
Respondent 122	7	7	5	7	6	6	7	5	6	7
Respondent 123	7	6	6	7	7	6	7	5	5	6
Respondent 124	2	6	5	7	7	5	4	5	5	5
Respondent 125	6	7	7	7	7	4	7	5	6	6
Respondent 126	7	6	7	7	7	7	7	5	6	7

Respondent 127	7	7	7	7	7	2	7	5	6	7
Respondent 128	7	6	7	7	7	7	7	6	6	7
Respondent 129	5	6	5	7	7	4	7	4	6	2
Respondent 130	6	5	6	7	7	4	6	7	6	7
Respondent 131	7	6	6	7	6	4	7	7	6	7
Respondent 132	7	7	7	7	7	4	7	7	6	7
Respondent 133	5	6	6	7	6	4	5	5	6	7
Respondent 134	6	7	5	7	6	4	6	6	2	7
Respondent 135	6	6	6	6	7	4	5	5	6	7
Respondent 136	7	7	7	7	6	7	7	7	7	7
Respondent 137	7	7	6	5	5	4	6	7	6	7
Respondent 138	7	6	6	7	7	4	7	5	2	7
Respondent 139	6	7	7	7	7	6	7	7	7	7
Respondent 140	6	6	7	7	7	6	7	7	6	7
Respondent 141	7	7	6	7	5	6	7	7	7	7
Respondent 142	7	6	7	7	7	7	7	7	7	7
Respondent 143	5	6	6	6	6	5	7	7	7	7
Respondent 144	7	7	7	6	6	6	7	7	7	7
Respondent 145	6	6	6	6	5	5	7	6	6	7
Respondent 146	6	6	7	5	5	6	6	6	7	7
Respondent 147	6	7	6	6	6	6	7	7	7	7
Respondent 148	7	6	7	7	6	7	7	7	7	6
Respondent 149	6	7	7	7	5	6	7	7	7	6
Respondent 150	6	7	7	7	6	7	7	7	7	6
Respondent 151	7	5	7	6	7	7	7	7	7	7
Respondent 152	7	7	6	6	5	6	7	7	7	7
Respondent 153	7	6	7	7	6	6	7	7	7	7
Respondent 154	6	7	7	7	5	6	7	7	7	7
Respondent 155	6	7	6	7	6	6	7	7	7	7
Respondent 156	6	5	7	6	5	6	7	7	7	7
Respondent 157	6	7	7	6	2	5	7	7	7	7
Respondent 158	7	6	7	7	6	7	7	7	7	7
Respondent 159	6	6	7	6	6	7	7	7	7	7
Respondent 160	6	6	7	7	6	7	7	7	7	7
Respondent 161	7	6	7	7	7	7	7	7	7	7
Respondent 162	7	7	7	7	6	7	7	7	7	7
Respondent 163	6	6	6	7	7	7	7	7	7	7
Respondent 164	6	7	6	6	6	7	7	7	7	7
Respondent 165	7	6	7	6	6	7	7	7	7	7
Respondent 166	6	7	6	6	6	7	7	7	7	7
Respondent 167	7	6	6	7	6	6	7	7	7	7
Respondent 168	6	7	5	6	6	6	7	7	7	7
Respondent 169	7	7	7	7	7	7	7	7	7	7
Respondent 170	7	7	7	6	6	6	7	7	7	7

Respondent 171	7	6	7	7	7	7	7	7	7	7
Respondent 172	6	5	6	6	6	6	6	6	6	6
Respondent 173	6	6	7	7	7	7	7	7	7	7
Respondent 174	5	7	6	5	2	5	6	6	7	6
Respondent 175	6	6	6	6	6	6	6	6	6	6
Respondent 176	6	6	7	6	6	6	7	6	7	7
Respondent 177	6	5	6	6	6	6	6	6	6	6
Respondent 178	7	7	7	7	7	7	7	7	7	7
Respondent 179	7	7	7	7	7	7	7	7	7	7
Respondent 180	6	5	6	6	6	6	6	6	7	6
Respondent 181	6	7	7	6	6	6	6	6	7	6
Respondent 182	7	5	7	7	7	7	7	7	7	7
Respondent 183	5	6	5	5	5	5	5	5	7	5
Respondent 184	6	6	5	5	5	5	6	6	7	6
Respondent 185	7	7	7	7	7	7	7	7	7	7
Respondent 186	6	6	6	6	6	6	6	6	6	6
Respondent 187	7	6	7	7	7	7	7	7	7	7
Respondent 188	5	7	6	5	5	5	6	6	6	6
Respondent 189	5	6	5	5	5	5	5	5	5	5
Respondent 190	6	7	6	6	6	6	7	7	7	7
Respondent 191	7	6	7	7	7	7	7	7	7	7
Respondent 192	5	7	7	5	6	7	6	5	7	7
Respondent 193	7	6	6	5	6	7	6	7	5	6
Respondent 194	7	7	7	7	7	6	7	7	7	7
Respondent 195	5	6	5	5	5	5	6	6	6	6
Respondent 196	7	4	7	7	7	7	7	7	7	7
Respondent 197	6	7	6	5	5	5	6	6	6	6
Respondent 198	7	6	7	7	7	7	7	7	7	7
Respondent 199	6	6	7	7	7	7	7	7	7	7
Respondent 200	7	7	7	7	7	7	7	7	7	7
Respondent 201	7	6	7	6	6	7	7	7	7	7
Respondent 202	7	7	7	7	6	6	7	7	7	7
Respondent 203	7	7	7	6	5	6	7	7	7	7
Respondent 204	6	6	6	7	6	6	7	7	7	7
Respondent 205	7	3	7	7	6	6	7	7	7	7
Respondent 206	7	6	7	7	6	6	7	7	7	7
Respondent 207	6	7	7	6	6	6	7	7	7	7
Respondent 208	6	6	7	7	5	6	7	7	7	7
Respondent 209	7	5	7	6	5	6	7	7	7	7
Respondent 210	7	6	7	6	5	7	7	7	7	7
Respondent 211	6	6	6	6	6	6	6	6	6	6
Respondent 212	6	7	6	6	6	6	6	6	6	6
Respondent 213	7	6	7	7	7	7	7	7	7	7
Respondent 214	7	5	7	7	7	7	7	7	7	7

Respondent 215	7	6	7	7	7	7	7	7	7	7
Respondent 216	5	6	6	5	5	5	6	6	6	5
Respondent 217	7	5	7	7	7	7	7	7	7	7
Respondent 218	6	6	6	6	6	6	6	6	6	6
Respondent 219	7	7	7	7	7	7	7	7	7	7
Respondent 220	7	6	7	7	7	7	7	7	7	7
Respondent 221	7	5	7	7	7	7	7	7	7	7
Respondent 222	6	6	6	6	6	6	6	6	6	6
Respondent 223	7	7	7	7	7	7	7	7	7	7
Respondent 224	5	6	5	6	6	6	6	6	6	5
Respondent 225	7	6	7	7	7	7	7	7	7	7
Respondent 226	7	7	7	7	7	7	7	7	7	7
Respondent 227	6	6	7	6	6	6	7	7	7	6
Respondent 228	7	6	7	7	7	7	7	7	7	7
Respondent 229	7	4	7	7	7	7	7	7	7	7
Respondent 230	6	6	6	7	7	7	7	7	7	6
Respondent 231	7	7	7	7	6	7	7	7	7	6
Respondent 232	5	6	6	5	2	4	5	5	5	2
Respondent 233	7	6	6	6	6	6	7	7	7	7
Respondent 234	7	6	7	7	7	7	7	7	7	7
Respondent 235	7	5	7	7	7	7	7	7	7	7
Respondent 236	6	6	7	7	7	4	7	7	7	7
Respondent 237	5	7	6	5	5	5	5	5	5	5
Respondent 238	6	6	6	5	6	6	6	6	6	6
Respondent 239	7	7	7	7	7	7	7	7	7	7
Respondent 240	6	7	6	6	6	6	6	6	6	6
Respondent 241	7	6	7	7	7	7	7	7	7	7
Respondent 242	7	6	7	6	6	6	7	7	7	7
Respondent 243	7	5	7	6	6	6	7	7	7	7
Respondent 244	7	7	7	7	6	7	7	7	7	7
Respondent 245	7	6	5	7	7	6	7	7	7	7
Respondent 246	2	6	7	6	6	6	6	6	6	7
Respondent 247	7	7	7	7	7	7	7	7	7	7
Respondent 248	7	6	7	7	7	7	7	7	7	7
Respondent 249	7	4	7	7	7	6	7	7	7	7
Respondent 250	6	6	6	6	6	6	6	6	6	6
Respondent 251	6	6	7	5	5	5	7	7	7	7
Respondent 252	6	5	6	5	5	5	6	6	6	6
Respondent 253	7	7	7	7	7	7	7	7	7	7
Respondent 254	7	6	7	7	7	7	7	7	7	7
Respondent 255	6	7	6	6	6	6	6	6	6	6
Respondent 256	6	6	6	6	6	6	6	6	6	6
Respondent 257	7	6	7	7	7	7	7	7	7	7
Respondent 258	5	6	6	5	5	5	7	7	7	7

Respondent 259	7	5	7	7	7	7	7	7	7	7
Respondent 260	6	6	6	6	6	6	7	7	7	7
Respondent 261	7	6	7	7	7	7	7	7	7	7
Respondent 262	6	7	6	6	5	5	6	6	6	6
Respondent 263	6	6	6	6	6	6	6	6	6	6
Respondent 264	7	3	7	7	7	7	7	7	7	7
Respondent 265	5	6	7	5	5	5	6	6	6	6
Respondent 266	6	6	6	6	6	6	6	6	6	6
Respondent 267	6	4	6	6	6	6	7	7	7	6
Respondent 268	7	6	7	7	7	7	7	7	7	7
Respondent 269	7	6	7	7	7	7	7	7	7	7
Respondent 270	2	7	5	2	2	2	5	5	5	2
Respondent 271	7	6	7	7	7	7	7	7	7	7
Respondent 272	6	7	6	6	6	6	6	6	6	6
Respondent 273	6	6	6	6	6	6	7	7	7	6
Respondent 274	7	6	7	7	7	7	7	7	7	7
Respondent 275	7	6	7	7	7	7	7	7	7	7
Respondent 276	7	5	7	7	7	7	7	7	7	7
Respondent 277	6	6	6	6	6	6	7	7	7	7
Respondent 278	5	4	6	5	5	5	7	7	7	7
Respondent 279	5	6	6	6	6	6	6	6	6	6
Respondent 280	7	7	7	7	7	7	7	7	7	7
Respondent 281	7	6	7	7	7	7	7	6	7	5
Respondent 282	7	6	7	7	6	7	7	7	7	7
Respondent 283	7	3	7	6	6	7	7	7	7	7
Respondent 284	6	6	6	6	5	6	7	7	7	7
Respondent 285	6	5	7	6	5	6	7	7	7	6
Respondent 286	7	6	7	6	5	6	7	7	7	7
Respondent 287	6	6	7	7	5	6	7	7	7	7
Respondent 288	6	7	6	6	5	6	7	7	7	7
Respondent 289	6	6	6	5	6	5	7	7	7	7
Respondent 290	6	6	7	6	5	6	7	7	7	7
Respondent 291	7	6	7	7	7	7	7	7	7	7
Respondent 292	7	7	7	6	6	6	7	7	7	7
Respondent 293	2	6	2	5	5	5	5	5	5	5
Respondent 294	6	5	6	6	6	6	6	6	6	6
Respondent 295	6	6	7	7	7	7	7	7	7	7
Respondent 296	7	6	7	7	7	7	7	7	7	7
Respondent 297	7	4	7	7	7	7	7	7	7	7
Respondent 298	7	6	5	7	7	7	6	7	6	7
Respondent 299	5	5	7	6	6	6	6	6	6	7
Respondent 300	7	6	7	6	7	6	6	6	6	6
Respondent 301	7	6	7	7	6	7	7	7	7	7
Respondent 302	6	7	7	7	7	7	7	7	7	7

Respondent 303	7	6	7	7	7	6	7	7	7	7
Respondent 304	6	6	7	7	7	7	7	7	7	7
Respondent 305	5	6	7	7	7	7	7	7	7	7
Respondent 306	7	2	7	7	7	7	7	7	7	7
Respondent 307	7	6		7	6	7	7	7	7	7
Respondent 308	7	3	7	7	7	7	7	7	7	6
Respondent 309	7	6	7	7	5	5	6	6	6	7
Respondent 310	6	4	5	6	6	6	6	7	6	6
Respondent 311	7	6	7	7	6	6	7	7	6	7
Respondent 312	6	4	6	6	6	6	6	6	5	6
Respondent 313	7	6	6	6	6	5	6	7	5	7
Respondent 314	7	5	7	7	7	5	7	7	5	7
Respondent 315	7	6	7	7	6	6	7	7	5	7
Respondent 316	7	5	7	7	7	6	7	7	6	7
Respondent 317	6	6	6	6	6	5	6	7	7	7
Respondent 318	6	7	6	7	7	6	7	7	7	6
Respondent 319	7	5	6	6	6	6	7	7	7	7
Respondent 320	7	6	7	6	6	5	6	6	6	6
Respondent 321	5	5	7	7	7	7	7	7	7	7
Respondent 322	7	6	7	7	6	6	7	7	5	6
Respondent 323	7	6	4	7	7	6	7	7	7	7
Respondent 324	7	6	7	7	7	7	7	7	7	7
Respondent 325	7	5	7	7	7	7	7	7	7	7
Respondent 326	5	6	7	7	7	7	7	7	7	7
Respondent 327	7	7	4	6	6	6	7	7	7	6
Respondent 328	6	6	7	7	7	6	7	7	5	7
Respondent 329	7	7	7	7	7	7	7	7	7	7
Respondent 330	7	6	7	7	7	7	7	7	7	7
Respondent 331	6	7	5	6	5	5	4	5	6	6
Respondent 332	7	6	6	7	6	7	6	6	7	6
Respondent 333	6	7	4	4	7	7	5	7	5	7
Respondent 334	7	6	6	6	7	6	7	6	6	6
Respondent 335	6	5	6	5	7	5	5	5	6	5
Respondent 336	7	6	5	4	5	4	3	4	5	4
Respondent 337	6	7	5	3	3	5	5	3	5	5
Respondent 338	7	6	4	7	7	6	7	7	6	7
Respondent 339	6	7	7	5	5	6	7	7	7	6
Respondent 340	7	6	6	7	7	7	5	7	6	6
Respondent 341	6	7	7	6	6	7	6	6	6	6
Respondent 342	7	6	5	7	6	7	7	7	6	7
Respondent 343	5	5	6	6	5	6	6	6	5	7
Respondent 344	6	6	5	5	6	6	6	6	6	6
Respondent 345	7	4	6	6	7	6	7	5	7	5
Respondent 346	7	6	5	7	6	7	7	5	6	6

Response to questionnaire (31-40)

	X31	X32	X33	X34	X35	X36	X37	X38	X39	X40
Respondent 1	6	2	6	7	6	7	7	7	7	7
Respondent 2	7	7	7	6	7	7	7	7	7	6
Respondent 3	6	7	6	7	7	6	7	4	7	7
Respondent 4	5	7	7	6	5	7	6	7	6	6
Respondent 5	5	7	7	7	7	7	7	7	7	7
Respondent 6	6	7	3	6	5	5	7	7	7	7
Respondent 7	5	7	6	7	2	7	6	6	5	2
Respondent 8	6	7	7	6	7	5	7	7	7	7
Respondent 9	7	7	5	5	7	7	7	7	7	7
Respondent 10	7	7	7	5	6	6	4	7	7	7
Respondent 11	7	7	6	6	7	7	7	6	5	6
Respondent 12	6	7	7	5	6	7	5	6	5	7
Respondent 13	7	7	4	6	7	6	7	2	7	7
Respondent 14	6	7	7	5	6	5	5	6	7	7
Respondent 15	2	7	6	7	7	7	5	6	7	7
Respondent 16	6	7	7	6	5	5	7	6	6	5
Respondent 17	6	6	4	7	7	6	7	5	6	5
Respondent 18	6	7	7	6	7	5	7	7	7	7
Respondent 19	5	5	6	4	6	7	7	7	7	7
Respondent 20	2	5	7	6	7	6	6	6	6	4
Respondent 21	7	7	3	5	5	7	7	7	7	7
Respondent 22	7	7	7	6	6	7	7	7	7	7
Respondent 23	7	7	6	4	6	7	7	6	7	6
Respondent 24	6	7	7	6	2	5	7	7	6	7
Respondent 25	7	7	5	3	4	7	7	7	7	6
Respondent 26	5	7	7	6	5	5	7	6	7	7
Respondent 27	7	7	6	4	7	7	7	6	6	7
Respondent 28	7	7	7	6	7	7	7	7	7	7
Respondent 29	7	7	7	7	7	7	7	6	7	7
Respondent 30	7	7	4	6	7	7	7	7	6	7
Respondent 31	7	7	7	7	6	6	7	6	7	7
Respondent 32	7	7	5	6	4	7	5	7	5	6
Respondent 33	6	7	7	6	7	7	7	6	6	7
Respondent 34	6	7	6	7	3	5	7	5	5	2
Respondent 35	6	7	7	6	5	7	5	4	5	4
Respondent 36	7	7	5	7	7	7	7	7	7	7
Respondent 37	7	5	7	6	7	7	2	6	5	7
Respondent 38	6	7	7	6	5	7	6	5	5	7

Respondent 39	5	7	7	7	7	6	5	6	7	7
Respondent 40	7	7	6	3	6	6	5	7	7	5
Respondent 41	7	7	7	5	7	7	7	7	7	7
Respondent 42	7	7	7	6	6	7	7	7	7	7
Respondent 43	7	7	7	5	4	7	7	7	6	7
Respondent 44	7	7	5	7	7	7	6	7	7	7
Respondent 45	7	7	7	3	7	7	7	5	6	7
Respondent 46	7	7	7	6	6	5	7	6	6	7
Respondent 47	7	7	6	4	7	7	5	7	7	7
Respondent 48	7	7	7	6	7	7	7	7	6	4
Respondent 49	7	7	7	7	7	7	7	7	7	7
Respondent 50	7	6	4	6	5	7	6	6	7	6
Respondent 51	7	7	7	6	7	6	7	7	7	7
Respondent 52	6	7	7	5	5	7	7	7	7	7
Respondent 53	6	7	5	6	5	6	7	7	7	6
Respondent 54	7	7	7	7	6	6	6	7	6	7
Respondent 55	5	5	7	6	7	5	7	7	6	5
Respondent 56	6	7	6	5	6	4	7	6	7	7
Respondent 57	6	7	4	6	5	7	7	7	7	7
Respondent 58	7	7	7	5	6	5	6	5	6	7
Respondent 59	7	6	6	6	7	7	6	7	7	6
Respondent 60	5	7	7	5	7	6	6	5	7	6
Respondent 61	7	7	7	6	6	6	7	7	5	7
Respondent 62	5	6	5	7	5	7	6	7	7	4
Respondent 63	6	7	7	6	5	7	7	7	6	6
Respondent 64	6	7	7	7	6	5	5	7	6	7
Respondent 65	2	7	5	6	5	6	2	2	6	4
Respondent 66	4	2	7	4	7	4	7	4	4	4
Respondent 67	7	7	7	6	6	4	7	7	7	7
Respondent 68	7	7	4	7	7	5	7	7	7	7
Respondent 69	7	7	7	7	7	7	7	7	7	6
Respondent 70	6	2	7	6	7	5	6	7	7	5
Respondent 71	7	5	5	6	6	6	7	6	6	7
Respondent 72	7	6	7	7	7	5	7	7	7	5
Respondent 73	6	7	6	6	5	5	7	6	5	7
Respondent 74	7	6	7	7	7	7	6	7	7	4
Respondent 75	6	7	6	5	7	6	6	5	7	7
Respondent 76	7	6	7	3	6	5	6	7	6	6
Respondent 77	2	7	5	5	7	5	7	7	7	7
Respondent 78	6	7	7	6	6	6	5	7	6	7
Respondent 79	6	7	7	5	6	7	6	7	5	6
Respondent 80	6	7	6	6	5	6	6	5	5	5
Respondent 81	7	7	7	3	5	6	5	6	7	4
Respondent 82	6	7	7	7	6	6	6	5	6	6

Respondent 83	6	7	5	6	6	7	6	6	7	7
Respondent 84	6	7	7	5	7	7	6	7	7	4
Respondent 85	6	7	6	5	8	7	7	5	6	7
Respondent 86	6	7	7	7	7	6	4	7	6	7
Respondent 87	7	7	7	5	6	5	7	7	7	5
Respondent 88	7	7	5	5	7	6	7	6	7	4
Respondent 89	6	5	7	7	7	6	6	6	7	6
Respondent 90	7	6	7	7	6	7	7	7	7	7
Respondent 91	6	7	6	4	6	7	6	6	6	7
Respondent 92	7	7	7	7	7	7	6	6	7	7
Respondent 93	7	7	7	5	6	6	6	7	6	6
Respondent 94	6	7	5	6	7	7	6	7	6	7
Respondent 95	5	7	7	7	6	6	7	6	7	7
Respondent 96	6	7	6	6	7	7	5	7	6	7
Respondent 97	6	7	5	7	5	5	4	7	6	4
Respondent 98	2	7	6	6	6	4	6	7	6	6
Respondent 99	7	6	7	7	7	4	5	5	5	2
Respondent 100	7	6	5	6	7	5	6	7	6	7
Respondent 101	6	7	5	7	6	7	6	6	6	7
Respondent 102	7	7	5	7	7	7	6	7	7	7
Respondent 103	7	7	5	6	6	6	6	7	6	7
Respondent 104	6	7	6	7	7	3	6	7	6	7
Respondent 105	5	7	7	7	6	6	7	7	7	7
Respondent 106	6	7	7	5	7	7	5	7	6	7
Respondent 107	6	7	6	6	4	5	4	7	6	4
Respondent 108	2	7	7	7	5	2	6	5	6	6
Respondent 109	7	6	5	5	7	5	5	7	6	2
Respondent 110	7	6	6	6	6	5	6	7	6	7
Respondent 111	7	7	6	6	6	5	6	5	6	5
Respondent 112	7	6	4	3	2	4	7	6	7	6
Respondent 113	5	5	7	6	5	7	7	5	6	6
Respondent 114	6	5	6	5	7	6	7	7	6	7
Respondent 115	7	7	7	6	7	7	7	7	7	7
Respondent 116	7	6	5	6	2	7	5	5	5	6
Respondent 117	6	7	6	7	2	5	5	5	6	6
Respondent 118	6	7	7	5	7	7	7	7	6	7
Respondent 119	7	7	5	6	7	7	7	6	6	7
Respondent 120	5	6	6	6	4	3	5	7	5	2
Respondent 121	7	7	7	5	7	6	7	6	7	7
Respondent 122	6	7	7	6	7	7	7	7	7	5
Respondent 123	7	7	5	6	7	7	7	6	7	7
Respondent 124	6	7	7	4	4	4	7	4	5	4
Respondent 125	7	7	7	6	5	5	7	5	7	7
Respondent 126	7	7	6	5	7	7	7	7	7	7

Respondent 127	7	7	7	6	7	7	7	7	7	7
Respondent 128	7	7	5	7	6	6	7	6	7	7
Respondent 129	6	7	6	7	7	5	7	6	7	7
Respondent 130	7	7	7	6	7	6	7	7	6	7
Respondent 131	6	7	7	5	7	7	6	7	7	7
Respondent 132	7	7	5	6	7	7	7	6	6	7
Respondent 133	6	7	7	6	5	6	7	7	6	7
Respondent 134	6	7	5	5	6	6	7	6	7	7
Respondent 135	6	7	5	7	5	6	6	6	7	4
Respondent 136	7	7	5	5	7	7	7	7	7	7
Respondent 137	7	7	5	6	5	5	7	7	7	4
Respondent 138	7	7	6	7	6	7	7	7	6	7
Respondent 139	7	6	7	6	6	7	7	7	6	7
Respondent 140	7	6	5	7	7	6	7	7	6	6
Respondent 141	7	7	6	6	7	7	6	7	7	7
Respondent 142	6	7	5	5	7	6	6	6	6	6
Respondent 143	7	6	6	7	6	6	6	6	7	6
Respondent 144	6	6	5	5	7	7	5	6	7	7
Respondent 145	6	6	6	6	6	6	7	7	6	5
Respondent 146	6	6	5	7	6	7	6	5	6	6
Respondent 147	6	6	6	6	7	6	5	7	7	6
Respondent 148	7	7	5	7	7	6	6	6	7	6
Respondent 149	7	7	7	7	7	6	6	7	6	6
Respondent 150	7	6	6	6	6	6	6	6	7	7
Respondent 151	7	7	5	7	7	6	6	6	7	7
Respondent 152	6	6	7	6	7	7	5	6	6	7
Respondent 153	6	6	6	6	6	6	6	6	7	7
Respondent 154	7	7	7	5	7	7	6	7	7	7
Respondent 155	6	6	5	6	7	6	5	7	7	6
Respondent 156	6	7	5	5	7	7	7	7	7	6
Respondent 157	6	6	5	5	5	7	6	6	7	6
Respondent 158	7	7	6	6	7	6	7	7	7	7
Respondent 159	7	7	7	5	5	6	7	7	7	6
Respondent 160	6	7	5	6	7	7	7	6	6	7
Respondent 161	7	7	6	5	7	7	6	7	5	7
Respondent 162	7	7	5	5	5	7	5	7	7	7
Respondent 163	7	6	6	6	7	6	5	7	6	7
Respondent 164	6	7	5	6	7	7	4	7	7	7
Respondent 165	6	7	6	6	5	7	4	6	7	7
Respondent 166	7	7	5	6	7	7	7	7	7	7
Respondent 167	6	7	7	7	5	7	7	7	7	6
Respondent 168	5	6	5	6	7	7	6	6	5	6
Respondent 169	6	6	7	7	5	6	5	6	6	5
Respondent 170	7	7	6	6	7	6	5	7	7	6

Respondent 171	7	7	7	6	7	7	7	7	7	7
Respondent 172	6	6	5	6	5	6	6	6	6	6
Respondent 173	7	7	7	6	4	7	7	7	7	7
Respondent 174	6	6	6	6	7	6	5	6	6	6
Respondent 175	6	6	7	4	6	6	6	6	6	6
Respondent 176	7	7	5	6	7	6	6	6	6	6
Respondent 177	6	6	6	4	6	6	6	6	6	6
Respondent 178	7	7	7	6	7	7	7	7	7	7
Respondent 179	7	7	5	4	7	7	7	6	7	7
Respondent 180	6	6	7	6	4	6	6	6	6	6
Respondent 181	6	6	7	3	6	6	6	6	6	6
Respondent 182	6	7	6	6	7	7	7	7	7	7
Respondent 183	5	5	7	6	5	6	6	6	6	6
Respondent 184	6	6	5	6	7	7	6	5	6	6
Respondent 185	7	7	7	6	4	7	7	7	7	7
Respondent 186	6	6	6	7	6	6	6	6	6	6
Respondent 187	7	7	7	6	7	7	7	7	7	7
Respondent 188	6	6	6	6	4	5	5	5	6	5
Respondent 189	5	5	7	6	5	5	5	5	5	5
Respondent 190	7	7	7	5	7	7	7	7	7	7
Respondent 191	7	7	6	4	7	7	7	7	7	7
Respondent 192	7	7	7	6	7	6	5	5	5	5
Respondent 193	7	6	7	7	7	7	7	7	7	7
Respondent 194	7	7	5	6	6	7	7	6	7	6
Respondent 195	6	6	7	7	6	6	5	5	5	5
Respondent 196	7	7	4	6	7	5	7	7	7	7
Respondent 197	6	6	7	7	6	6	5	5	6	5
Respondent 198	7	7	5	6	7	7	7	7	7	7
Respondent 199	7	7	7	6	7	7	6	6	6	6
Respondent 200	7	7	6	6	7	7	7	7	7	7
Respondent 201	6	6	4	7	4	5	6	7	7	6
Respondent 202	6	6	5	6	7	7	7	6	7	7
Respondent 203	7	7	7	7	4	6	6	6	6	6
Respondent 204	7	7	5	6	7	5	6	7	7	7
Respondent 205	6	6	7	7	7	7	7	6	6	6
Respondent 206	7	7	5	4	5	6	6	6	7	6
Respondent 207	6	6	4	6	7	6	7	6	7	7
Respondent 208	6	6	7	3	5	5	6	6	4	7
Respondent 209	7	6	5	6	7	6	6	7	7	7
Respondent 210	6	6	7	6	6	7	7	7	7	6
Respondent 211	6	6	6	6	5	6	6	6	6	6
Respondent 212	6	6	4	5	6	5	6	6	6	6
Respondent 213	7	7	7	6	7	7	7	7	7	7
Respondent 214	7	7	5	3	7	7	7	7	7	7

Respondent 215	7	7	3	6	5	7	7	7	7	7
Respondent 216	5	5	7	5	6	5	6	5	6	6
Respondent 217	7	7	5	6	7	7	7	7	7	7
Respondent 218	6	6	7	7	6	6	6	6	6	6
Respondent 219	7	7	5	6	5	7	7	7	7	7
Respondent 220	7	7	3	7	7	5	7	7	7	7
Respondent 221	7	7	5	6	7	7	7	7	7	7
Respondent 222	6	6	7	6	6	6	6	6	6	6
Respondent 223	7	7	5	5	7	7	7	7	7	7
Respondent 224	5	5	7	6	5	6	6	6	6	6
Respondent 225	7	7	5	5	7	5	7	7	7	7
Respondent 226	7	7	7	5	7	7	7	7	7	7
Respondent 227	6	6	3	6	4	5	6	6	6	6
Respondent 228	7	7	6	5	7	7	7	7	7	7
Respondent 229	7	7	7	7	7	5	7	7	7	7
Respondent 230	6	6	7	6	5	7	6	6	6	6
Respondent 231	7	7	3	7	7	7	7	7	6	7
Respondent 232	2	2	7	6	6	5	5	5	2	5
Respondent 233	7	6	7	7	4	5	6	7	6	6
Respondent 234	7	7	5	7	7	6	7	7	7	7
Respondent 235	7	7	7	6	7	7	7	7	7	7
Respondent 236	7	7	4	7	5	6	5	4	7	6
Respondent 237	5	5	7	6	5	7	5	5	6	6
Respondent 238	6	6	5	7	6	6	6	6	6	7
Respondent 239	7	7	5	7	7	7	7	7	6	6
Respondent 240	6	6	7	7	6	7	6	6	6	6
Respondent 241	7	7	5	7	7	7	7	6	7	7
Respondent 242	7	7	7	7	7	7	7	7	7	6
Respondent 243	7	6	6	6	7	7	6	6	7	7
Respondent 244	7	7	7	7	7	5	6	5	7	7
Respondent 245	7	6	5	6	7	7	6	7	6	5
Respondent 246	7	6	6	7	6	5	7	5	7	7
Respondent 247	7	7	7	6	7	7	7	7	7	7
Respondent 248	7	7	6	6	6	7	7	7	7	7
Respondent 249	7	7	7	5	5	6	7	7	7	7
Respondent 250	6	6	5	6	6	6	6	6	6	6
Respondent 251	7	7	7	4	7	7	5	5	6	7
Respondent 252	6	6	5	3	7	6	6	6	6	7
Respondent 253	7	7	7	6	7	7	7	7	7	7
Respondent 254	7	7	5	6	5	7	7	7	7	7
Respondent 255	6	6	4	5	6	6	6	6	6	6
Respondent 256	6	6	7	6	6	6	6	6	6	6
Respondent 257	7	7	7	5	4	7	7	7	7	7
Respondent 258	7	7	7	6	7	5	5	5	6	7

Respondent 259	7	7	7	5	7	7	7	7	7	7
Respondent 260	7	7	6	6	4	6	5	6	6	7
Respondent 261	7	7	7	6	7	7	7	7	7	7
Respondent 262	6	6	7	5	6	6	5	5	6	6
Respondent 263	6	6	6	7	5	6	5	6	6	6
Respondent 264	7	7	7	7	7	7	7	7	7	7
Respondent 265	6	6	4	6	5	6	6	6	6	6
Respondent 266	6	6	7	7	6	6	6	6	6	6
Respondent 267	6	6	7	6	7	7	6	6	6	6
Respondent 268	7	7	4	7	5	7	7	7	7	7
Respondent 269	7	7	7	4	7	7	7	7	7	7
Respondent 270	2	2	5	4	5	2	5	5	5	5
Respondent 271	7	7	7	4	7	7	7	7	7	7
Respondent 272	6	6	6	6	6	6	6	6	6	6
Respondent 273	6	6	7	6	7	7	6	6	6	6
Respondent 274	7	7	5	6	7	7	7	7	7	7
Respondent 275	7	7	5	6	7	7	7	7	7	7
Respondent 276	7	7	6	6	7	7	7	7	7	7
Respondent 277	7	7	5	7	7	6	5	5	5	6
Respondent 278	7	7	6	6	7	6	5	5	5	7
Respondent 279	6	6	7	7	6	7	6	6	6	6
Respondent 280	7	7	6	6	7	7	7	7	7	7
Respondent 281	6	7	7	6	7	6	7	7	7	7
Respondent 282	7	7	7	4	7	5	5	6	7	7
Respondent 283	6	7	5	6	6	6	6	7	7	7
Respondent 284	7	6	7	4	6	7	7	6	7	7
Respondent 285	5	6	5	6	7	6	7	7	7	6
Respondent 286	6	6	5	3	6	6	6	7	6	6
Respondent 287	6	6	5	5	7	7	6	6	6	7
Respondent 288	6	6	5	6	7	6	6	6	6	7
Respondent 289	7	7	5	3	6	6	6	7	6	6
Respondent 290	6	7	5	6	7	7	5	6	6	5
Respondent 291	7	7	7	4	7	7	7	7	6	6
Respondent 292	7	7	6	6	6	7	7	7	7	7
Respondent 293	5	5	7	3	5	7	7	6	7	6
Respondent 294	6	6	6	6	6	5	6	6	6	6
Respondent 295	7	7	5	6	7	7	7	7	7	7
Respondent 296	7	7	7	6	6	7	7	7	7	7
Respondent 297	7	7	5	6	7	7	7	6	7	7
Respondent 298	7	7	7	6	6	7	7	7	7	7
Respondent 299	6	6	5	7	7	7	7	7	7	7
Respondent 300	6	6	7	6	7	7	6	6	6	6
Respondent 301	7	7	4	7	6	6	7	7	7	5
Respondent 302	7	7	7	6	7	7	7	7	7	7

Respondent 303	7	7	7	6	6	7	7		6	7
Respondent 304	7	7	6	7	7	7	7	7	7	7
Respondent 305	7	7	7	6	6	7	7	7	7	5
Respondent 306	7	7	4	6	7	7	6	7	7	7
Respondent 307	7	7	7	7	6	6	7	7	7	7
Respondent 308	7	7	7	6	7	7	7	7	7	7
Respondent 309	6	6	3	5	5	6	6	4	5	5
Respondent 310	6	6	7	6	6	7	5	6	5	6
Respondent 311	7	7	7	6	7	7	7	7	7	7
Respondent 312	7	7	7	7	7	6	6	6	6	6
Respondent 313	7	7	7	6	5	7	7	6	7	6
Respondent 314	7	7	4	5	7	7	7	7	7	7
Respondent 315	7	7	7	6	7	6	7	7	7	7
Respondent 316	7	7	5	5	5	7	5	6	6	6
Respondent 317	7	7	7	6	7	7	7	7	7	7
Respondent 318	7	6	6	6	7	7	6	6	5	7
Respondent 319	7	7	7	6	5	7	6	6	7	7
Respondent 320	7	7	5	6	6	5	6	6	5	7
Respondent 321	7	7	7	6	7	6	7	6	7	6
Respondent 322	6	7	7	5	5	7	7	6	7	7
Respondent 323	7	7	5	6	7	6	6	7	7	7
Respondent 324	7	7	7	5	7	7	7	7	5	7
Respondent 325	7	7	5	6	5	7	7	7	7	6
Respondent 326	7	7	7	6	7	7	7	7	7	7
Respondent 327	7	6	6	5	6	6	7	6	5	7
Respondent 328	7	7	7	6	5	7	6	7	7	6
Respondent 329	7	7	7	7	7	7	7	7	7	7
Respondent 330	7	7	5	6	5	7	7	7	7	7
Respondent 331	6	6	7	6	6	6	6	7	6	5
Respondent 332	7	7	7	6	7	6	7	6	7	6
Respondent 333	5	5	6	6	5	5	6	7	7	6
Respondent 334	7	7	7	7	7	6	7	6	5	3
Respondent 335	6	7	5	6	5	7	6	6	6	4
Respondent 336	4	5	6	6	5	6	7	7	5	5
Respondent 337	6	3	7	7	4	7	6	7	6	6
Respondent 338	7	7	7	6	5	5	7	6	7	7
Respondent 339	5	4	5	6	4	4	6	7	6	6
Respondent 340	7	6	7	5	6	4	7	7	5	7
Respondent 341	6	7	6	6	7	7	6	6	5	6
Respondent 342	7	6	7	6	5	7	6	7	6	7
Respondent 343	6	7	6	5	3	6	7	7	7	7
Respondent 344	7	6	7	6	6	7	6	7	6	7
Respondent 345	6	7	5	7	5	7	5	6	7	5
Respondent 346	7	7	7	6	6	5	6	5	6	6

Response to questionnaire (41-50)

	X41	X42	X43	X44	X45	X46	X47	X48	X49	X50
Respondent 1	7	6	4	7	6	4	7	4	4	7
Respondent 2	7	5	5	7	5	5	6	5	5	6
Respondent 3	6	7	7	6	7	7	7	7	7	7
Respondent 4	7	5	6	7	5	6	5	5	6	5
Respondent 5	7	4	6	7	4	6	7	4	6	7
Respondent 6	6	7	6	6	7	6	5	3	6	5
Respondent 7	4	6	5	4	6	5	7	2	5	7
Respondent 8	7	5	7	7	5	7	6	4	7	4
Respondent 9	5	5	2	5	5	7	5	5	6	5
Respondent 10	6	5	5	6	5	6	7	7	7	7
Respondent 11	7	6	4	7	4	7	5	6	5	7
Respondent 12	7	5	5	6	5	7	4	6	7	6
Respondent 13	6	7	7	7	7	6	7	6	5	7
Respondent 14	7	5	6	5	5	4	6	5	7	7
Respondent 15	7	4	6	7	4	7	5	7	7	6
Respondent 16	6	7	6	5	3	5	7	6	4	4
Respondent 17	4	6	5	7	2	6	7	5	5	7
Respondent 18	7	5	7	7	5	7	6	7	7	5
Respondent 19	5	5	2	6	7	7	7	5	6	6
Respondent 20	6	5	5	7	4	6	7	4	6	7
Respondent 21	7	6	4	7	4	7	6	7	6	7
Respondent 22	7	5	5	6	5	7	4	6	5	7
Respondent 23	6	7	7	7	7	7	7	5	7	6
Respondent 24	7	5	6	5	5	6	5	5	2	7
Respondent 25	7	4	6	7	4	7	5	6	5	7
Respondent 26	6	7	6	5	3	7	4	6	7	6
Respondent 27	4	6	5	7	2	6	7	6	5	4
Respondent 28	7	5	7	7	5	4	6	5	7	7
Respondent 29	5	5	2	6	7	7	5	7	7	5
Respondent 30	6	5	5	7	4	5	5	2	6	6
Respondent 31	4	6	5	7	2	6	5	5	7	6
Respondent 32	7	5	7	7	7	6	4	7	4	5
Respondent 33	5	5	2	6	7	5	5	6	5	7
Respondent 34	6	5	5	7	6	7	7	7	7	2
Respondent 35	7	6	4	7	7	5	6	5	5	7
Respondent 36	7	5	5	6	7	4	6	7	4	7
Respondent 37	6	7	7	7	6	7	6	5	3	6
Respondent 38	7	5	6	5	4	6	5	7	2	7

Respondent 39	7	4	6	7	7	5	7	7	5	7
Respondent 40	6	7	6	5	5	5	2	6	7	6
Respondent 41	4	6	5	7	6	5	5	7	4	4
Respondent 42	7	5	7	7	5	7	6	4	7	7
Respondent 43	5	5	2	6	7	7	5	5	6	5
Respondent 44	6	5	5	7	4	6	7	7	7	6
Respondent 45	7	6	4	7	4	7	5	6	5	5
Respondent 46	7	5	5	6	5	7	4	6	7	7
Respondent 47	4	6	5	7	2	6	7	6	5	4
Respondent 48	7	5	7	7	5	4	6	5	7	7
Respondent 49	5	5	2	6	7	7	5	7	7	5
Respondent 50	6	5	5	7	4	5	5	2	6	6
Respondent 51	4	6	5	7	2	6	5	5	7	6
Respondent 52	7	5	7	7	7	6	4	7	4	5
Respondent 53	5	5	2	6	7	5	5	6	5	7
Respondent 54	6	5	5	7	6	7	7	7	7	2
Respondent 55	7	6	4	7	7	5	6	5	5	7
Respondent 56	7	5	5	6	7	4	6	7	4	7
Respondent 57	6	7	7	7	6	7	6	5	3	6
Respondent 58	7	5	6	5	4	6	5	7	2	7
Respondent 59	7	4	6	7	7	5	7	7	5	7
Respondent 60	6	7	6	5	5	5	2	6	7	6
Respondent 61	4	6	5	7	6	5	5	7	4	4
Respondent 62	7	5	7	7	5	7	6	4	7	7
Respondent 63	5	5	2	6	7	7	5	5	6	5
Respondent 64	6	5	5	7	4	6	7	7	7	6
Respondent 65	4	6	5	7	2	6	7	6	5	4
Respondent 66	7	5	7	7	5	4	6	5	7	7
Respondent 67	5	5	2	6	7	7	5	7	7	5
Respondent 68	6	5	5	7	4	5	5	2	6	6
Respondent 69	4	6	5	7	2	6	5	5	7	6
Respondent 70	7	5	7	7	7	6	4	7	4	5
Respondent 71	5	5	2	6	7	5	5	6	5	7
Respondent 72	6	5	5	7	6	7	7	7	7	2
Respondent 73	7	6	4	7	7	5	6	5	5	7
Respondent 74	7	5	5	6	7	4	6	7	4	7
Respondent 75	6	7	7	7	6	7	6	5	3	6
Respondent 76	7	5	6	5	4	6	5	7	2	7
Respondent 77	7	4	6	7	7	5	7	7	5	7
Respondent 78	6	7	6	5	5	5	2	6	7	6
Respondent 79	4	6	5	7	6	5	5	7	4	4
Respondent 80	7	5	7	7	5	7	6	4	7	7
Respondent 81	5	5	2	6	7	7	5	5	6	5
Respondent 82	6	5	5	7	4	6	7	7	7	6

Respondent 83	7	6	4	7	4	7	5	6	5	5
Respondent 84	7	5	7	7	5	7	6	4	7	7
Respondent 85	5	5	2	6	7	7	5	5	6	5
Respondent 86	6	5	5	7	4	6	7	7	7	6
Respondent 87	7	6	4	7	4	7	5	6	5	5
Respondent 88	4	6	5	7	2	6	7	6	5	4
Respondent 89	7	5	7	7	5	4	6	5	7	7
Respondent 90	5	5	2	6	7	7	5	7	7	5
Respondent 91	6	5	5	7	4	5	5	2	6	6
Respondent 92	4	6	5	7	2	6	5	5	7	6
Respondent 93	7	5	7	7	7	6	4	7	4	5
Respondent 94	5	5	2	6	7	5	5	6	5	7
Respondent 95	6	5	5	7	6	7	7	7	7	2
Respondent 96	7	6	4	7	7	5	6	5	5	7
Respondent 97	7	5	5	6	7	4	6	7	4	7
Respondent 98	6	7	7	7	6	7	6	5	3	6
Respondent 99	7	5	6	5	4	6	5	7	2	7
Respondent 100	7	4	6	7	7	5	7	7	5	7
Respondent 101	6	7	6	5	5	5	2	6	7	6
Respondent 102	4	6	5	7	6	5	5	7	4	4
Respondent 103	7	5	7	7	5	7	6	4	7	7
Respondent 104	5	5	2	6	7	7	5	5	6	5
Respondent 105	6	5	5	7	4	6	7	7	7	6
Respondent 106	7	6	4	7	4	7	5	6	5	5
Respondent 107	5	5	2	6	7	7	5	7	7	5
Respondent 108	6	5	5	7	4	5	5	2	6	6
Respondent 109	4	6	5	7	2	6	5	5	7	6
Respondent 110	7	5	7	7	7	6	4	7	4	5
Respondent 111	5	5	2	6	7	5	5	6	5	7
Respondent 112	6	5	5	7	6	7	7	7	7	2
Respondent 113	7	6	4	7	7	5	6	5	5	7
Respondent 114	7	5	5	6	7	4	6	7	4	7
Respondent 115	6	7	7	7	6	7	6	5	3	6
Respondent 116	7	5	6	5	4	6	5	7	2	7
Respondent 117	7	4	6	7	7	5	7	7	5	7
Respondent 118	6	7	6	5	5	5	2	6	7	6
Respondent 119	4	6	5	7	6	5	5	7	4	4
Respondent 120	7	5	7	7	5	7	6	4	7	7
Respondent 121	5	5	2	6	7	7	5	5	6	5
Respondent 122	6	5	5	7	4	6	7	7	7	6
Respondent 123	4	6	5	7	2	6	7	6	5	4
Respondent 124	7	5	7	7	5	4	6	5	7	7
Respondent 125	5	5	2	6	7	7	5	7	7	5
Respondent 126	6	5	5	7	4	5	5	2	6	6

Respondent 127	4	6	5	7	2	6	5	5	7	6
Respondent 128	7	5	7	7	7	6	4	7	4	5
Respondent 129	5	5	2	6	7	5	5	6	5	7
Respondent 130	6	5	5	7	6	7	7	7	7	2
Respondent 131	7	6	4	7	7	5	6	5	5	7
Respondent 132	7	5	5	6	7	4	6	7	4	7
Respondent 133	6	7	7	7	6	7	6	5	3	6
Respondent 134	7	5	6	5	4	6	5	7	2	7
Respondent 135	7	4	6	7	7	5	7	7	5	7
Respondent 136	6	7	6	5	5	5	2	6	7	6
Respondent 137	4	6	5	7	6	5	5	7	4	4
Respondent 138	7	5	7	7	5	7	6	4	7	7
Respondent 139	5	5	2	6	7	7	5	5	6	5
Respondent 140	6	5	5	7	4	6	7	7	7	6
Respondent 141	7	6	4	7	4	7	5	6	5	5
Respondent 142	4	6	5	7	2	6	5	5	7	6
Respondent 143	7	5	7	7	7	6	4	7	4	5
Respondent 144	5	5	2	6	7	5	5	6	5	7
Respondent 145	6	5	5	7	6	7	7	7	7	2
Respondent 146	7	6	4	7	7	5	6	5	5	7
Respondent 147	7	5	5	6	7	4	6	7	4	7
Respondent 148	6	7	7	7	6	7	6	5	3	6
Respondent 149	7	5	6	5	4	6	5	7	2	7
Respondent 150	7	4	6	7	7	5	7	7	5	7
Respondent 151	6	7	6	5	5	5	2	6	7	6
Respondent 152	4	6	5	7	2	6	7	6	5	4
Respondent 153	7	5	7	7	5	4	6	5	7	7
Respondent 154	5	5	2	6	7	7	5	7	7	5
Respondent 155	6	5	5	7	4	5	5	2	6	6
Respondent 156	4	6	5	7	2	6	5	5	7	6
Respondent 157	7	5	7	7	7	6	4	7	4	5
Respondent 158	5	5	2	6	7	5	5	6	5	7
Respondent 159	6	5	5	7	6	7	7	7	7	2
Respondent 160	7	6	4	7	7	5	6	5	5	7
Respondent 161	7	5	5	6	7	4	6	7	4	7
Respondent 162	6	7	7	7	6	7	6	5	3	6
Respondent 163	7	5	6	5	4	6	5	7	2	7
Respondent 164	7	4	6	7	7	5	7	7	5	7
Respondent 165	6	7	6	5	5	5	2	6	7	6
Respondent 166	4	6	5	7	6	5	5	7	4	4
Respondent 167	7	5	7	7	5	7	6	4	7	7
Respondent 168	5	5	2	6	7	7	5	5	6	5
Respondent 169	6	5	5	7	4	6	7	7	7	6
Respondent 170	7	6	4	7	4	7	5	6	5	5

Respondent 171	6	5	5	7	6	7	7	7	7	2
Respondent 172	7	6	4	7	7	5	6	5	5	7
Respondent 173	7	5	5	6	7	4	6	7	4	7
Respondent 174	6	7	7	7	6	7	6	5	3	6
Respondent 175	7	5	6	5	4	6	5	7	2	7
Respondent 176	4	6	5	7	2	6	7	6	5	4
Respondent 177	7	5	7	7	5	4	6	5	7	7
Respondent 178	5	5	2	6	7	7	5	7	7	5
Respondent 179	6	5	5	7	4	5	5	2	6	6
Respondent 180	4	6	5	7	2	6	5	5	7	6
Respondent 181	7	5	7	7	7	6	4	7	4	5
Respondent 182	5	5	2	6	7	5	5	6	5	7
Respondent 183	6	5	5	7	6	7	7	7	7	2
Respondent 184	7	6	4	7	7	5	6	5	5	7
Respondent 185	7	5	5	6	7	4	6	7	4	7
Respondent 186	6	7	7	7	6	7	6	5	3	6
Respondent 187	7	5	6	5	4	6	5	7	2	7
Respondent 188	4	6	5	7	2	6	7	6	5	4
Respondent 189	7	5	7	7	5	4	6	5	7	7
Respondent 190	5	5	2	6	7	7	5	7	7	5
Respondent 191	6	5	5	7	4	5	5	2	6	6
Respondent 192	4	6	5	7	2	6	5	5	7	6
Respondent 193	7	5	7	7	7	6	4	7	4	5
Respondent 194	5	5	2	6	7	5	5	6	5	7
Respondent 195	6	5	5	7	6	7	7	7	7	2
Respondent 196	7	6	4	7	7	5	6	5	5	7
Respondent 197	7	5	5	6	7	4	6	7	4	7
Respondent 198	6	7	7	7	6	7	6	5	3	6
Respondent 199	7	5	6	5	4	6	5	7	2	7
Respondent 200	7	4	6	7	7	5	7	7	5	7
Respondent 201	6	7	6	5	5	5	2	6	7	6
Respondent 202	4	6	5	7	6	5	5	7	4	4
Respondent 203	7	5	7	7	5	7	6	4	7	7
Respondent 204	5	5	2	6	7	7	5	5	6	5
Respondent 205	6	5	5	7	4	6	7	7	7	6
Respondent 206	7	6	4	7	4	7	5	6	5	5
Respondent 207	7	5	6	5	4	6	5	7	2	7
Respondent 208	7	4	6	7	7	5	7	7	5	7
Respondent 209	6	7	6	5	5	5	2	6	7	6
Respondent 210	4	6	5	7	2	6	7	6	5	4
Respondent 211	7	5	7	7	5	4	6	5	7	7
Respondent 212	5	5	2	6	7	7	5	7	7	5
Respondent 213	6	5	5	7	4	5	5	2	6	6
Respondent 214	4	6	5	7	2	6	5	5	7	6

Respondent 215	7	5	7	7	7	6	4	7	4	5
Respondent 216	5	5	2	6	7	5	5	6	5	7
Respondent 217	6	5	5	7	6	7	7	7	7	2
Respondent 218	7	6	4	7	7	5	6	5	5	7
Respondent 219	7	5	5	6	7	4	6	7	4	7
Respondent 220	6	7	7	7	6	7	6	5	3	6
Respondent 221	7	5	6	5	4	6	5	7	2	7
Respondent 222	7	4	6	7	7	5	7	7	5	7
Respondent 223	6	7	6	5	5	5	2	6	7	6
Respondent 224	4	6	5	7	6	5	5	7	4	4
Respondent 225	7	5	7	7	5	7	6	4	7	7
Respondent 226	5	5	2	6	7	7	5	5	6	5
Respondent 227	6	5	5	7	4	6	7	7	7	6
Respondent 228	7	6	4	7	4	7	5	6	5	5
Respondent 229	7	5	5	6	7	4	6	7	4	7
Respondent 230	6	7	7	7	6	7	6	5	3	6
Respondent 231	7	5	6	5	4	6	5	7	2	7
Respondent 232	7	4	6	7	7	5	7	7	5	7
Respondent 233	6	7	6	5	5	5	2	6	7	6
Respondent 234	4	6	5	7	2	6	7	6	5	4
Respondent 235	7	5	7	7	5	4	6	5	7	7
Respondent 236	5	5	2	6	7	7	5	7	7	5
Respondent 237	6	5	5	7	4	5	5	2	6	6
Respondent 238	4	6	5	7	2	6	5	5	7	6
Respondent 239	7	5	7	7	7	6	4	7	4	5
Respondent 240	5	5	2	6	7	5	5	6	5	7
Respondent 241	6	5	5	7	6	7	7	7	7	2
Respondent 242	7	6	4	7	7	5	6	5	5	7
Respondent 243	7	5	5	6	7	4	6	7	4	7
Respondent 244	6	7	7	7	6	7	6	5	3	6
Respondent 245	7	5	6	5	4	6	5	7	2	7
Respondent 246	7	4	6	7	7	5	7	7	5	7
Respondent 247	6	7	6	5	5	5	2	6	7	6
Respondent 248	4	6	5	7	6	5	5	7	4	4
Respondent 249	7	5	7	7	5	7	6	4	7	7
Respondent 250	5	5	2	6	7	7	5	5	6	5
Respondent 251	6	5	5	7	4	6	7	7	7	6
Respondent 252	7	6	4	7	4	7	5	6	5	5
Respondent 253	5	5	2	6	7	7	5	7	7	5
Respondent 254	6	5	5	7	4	5	5	2	6	6
Respondent 255	4	6	5	7	2	6	5	5	7	6
Respondent 256	7	5	7	7	7	6	4	7	4	5
Respondent 257	5	5	2	6	7	5	5	6	5	7
Respondent 258	6	5	5	7	6	7	7	7	7	2

Respondent 259	7	6	4	7	7	5	6	5	5	7
Respondent 260	4	6	5	7	2	6	7	6	5	4
Respondent 261	7	5	7	7	5	4	6	5	7	7
Respondent 262	5	5	2	6	7	7	5	7	7	5
Respondent 263	6	5	5	7	4	5	5	2	6	6
Respondent 264	4	6	5	7	2	6	5	5	7	6
Respondent 265	7	5	7	7	7	6	4	7	4	5
Respondent 266	5	5	2	6	7	5	5	6	5	7
Respondent 267	6	5	5	7	6	7	7	7	7	2
Respondent 268	7	6	4	7	7	5	6	5	5	7
Respondent 269	7	5	5	6	7	4	6	7	4	7
Respondent 270	6	7	7	7	6	7	6	5	3	6
Respondent 271	7	5	6	5	4	6	5	7	2	7
Respondent 272	7	4	6	7	7	5	7	7	5	7
Respondent 273	6	7	6	5	5	5	2	6	7	6
Respondent 274	4	6	5	7	6	5	5	7	4	4
Respondent 275	7	5	7	7	5	7	6	4	7	7
Respondent 276	5	5	2	6	7	7	5	5	6	5
Respondent 277	6	5	5	7	4	6	7	7	7	6
Respondent 278	7	6	4	7	4	7	5	6	5	5
Respondent 279	7	5	7	7	5	7	6	4	7	4
Respondent 280	5	5	2	5	5	7	5	5	6	5
Respondent 281	6	5	5	6	5	6	7	7	7	7
Respondent 282	7	6	4	7	4	7	5	6	5	7
Respondent 283	7	5	5	6	5	7	4	6	7	6
Respondent 284	6	7	7	7	7	6	7	6	5	7
Respondent 285	7	5	6	5	5	4	6	5	7	7
Respondent 286	7	4	6	7	4	7	5	7	7	6
Respondent 287	6	7	6	5	3	5	7	6	4	4
Respondent 288	4	6	5	7	2	6	7	5	5	7
Respondent 289	7	5	7	7	5	7	6	7	7	5
Respondent 290	7	6	4	7	6	4	7	4	4	7
Respondent 291	7	5	5	7	5	5	6	5	5	6
Respondent 292	6	7	7	6	7	7	7	7	7	7
Respondent 293	7	5	6	7	5	6	5	5	6	5
Respondent 294	7	4	6	7	4	6	7	4	6	7
Respondent 295	6	7	6	6	7	6	5	3	6	5
Respondent 296	4	6	5	4	6	5	7	2	5	7
Respondent 297	7	5	7	7	5	7	6	4	7	4
Respondent 298	5	5	2	5	5	7	5	5	6	5
Respondent 299	6	5	5	6	5	6	7	7	7	7
Respondent 300	7	6	4	7	4	7	5	6	5	7
Respondent 301	7	5	5	6	5	7	4	6	7	6
Respondent 302	6	7	7	7	7	6	7	6	5	7

Respondent 303	7	5	6	5	5	4	6	5	7	7
Respondent 304	7	4	6	7	4	7	5	7	7	6
Respondent 305	6	7	6	5	3	5	7	6	4	4
Respondent 306	4	6	5	7	2	6	7	5	5	7
Respondent 307	7	5	7	7	5	7	6	7	7	5
Respondent 308	5	5	2	6	7	7	7	5	6	6
Respondent 309	6	5	5	7	4	6	7	4	6	7
Respondent 310	7	6	4	7	4	7	6	7	6	7
Respondent 311	7	5	5	6	5	7	4	6	5	7
Respondent 312	6	7	7	7	7	7	7	5	7	6
Respondent 313	6	7	7	6	7	7	7	7	7	7
Respondent 314	7	5	6	7	5	6	5	5	6	5
Respondent 315	7	4	6	7	4	6	7	4	6	7
Respondent 316	6	7	6	6	7	6	5	3	6	5
Respondent 317	4	6	5	4	6	5	7	2	5	7
Respondent 318	7	5	7	7	5	7	6	4	7	4
Respondent 319	5	5	2	5	5	7	5	5	6	5
Respondent 320	6	5	5	6	5	6	7	7	7	7
Respondent 321	7	6	4	7	4	7	5	6	5	7
Respondent 322	7	5	5	6	5	7	4	6	7	6
Respondent 323	6	7	7	7	7	6	7	6	5	7
Respondent 324	7	5	6	5	5	4	6	5	7	7
Respondent 325	7	4	6	7	4	7	5	7	7	6
Respondent 326	6	7	6	5	3	5	7	6	4	4
Respondent 327	4	6	5	7	2	6	7	5	5	7
Respondent 328	7	5	7	7	5	7	6	7	7	5
Respondent 329	5	5	2	6	7	7	7	5	6	6
Respondent 330	6	5	5	7	4	6	7	4	6	7
Respondent 331	7	6	4	7	4	7	6	7	6	7
Respondent 332	7	5	5	6	5	7	4	6	5	7
Respondent 333	6	7	7	7	7	7	7	5	7	6
Respondent 334	7	5	7	7	5	7	6	4	7	4
Respondent 335	5	5	2	5	5	7	5	5	6	5
Respondent 336	6	5	5	6	5	6	7	7	7	7
Respondent 337	7	6	4	7	4	7	5	6	5	7
Respondent 338	7	5	5	6	5	7	4	6	7	6
Respondent 339	7	5	7	7	5	7	6	4	7	4
Respondent 340	5	5	2	5	5	7	5	5	6	5
Respondent 341	6	5	5	6	5	6	7	7	7	7
Respondent 342	7	6	4	7	4	7	5	6	5	7
Respondent 343	7	5	5	6	5	7	4	6	7	6
Respondent 344	6	7	7	7	7	6	7	6	5	7
Respondent 345	7	5	6	5	5	4	6	5	7	7
Respondent 346	7	4	6	7	4	7	5	7	7	6

Response to questionnaire (51-60)

	X51	X52	X53	X54	X55	X56	X57	X58	X59	X60
Respondent 1	4	7	6	4	7	7	6	4	7	4
Respondent 2	5	7	5	5	6	7	5	5	6	5
Respondent 3	7	6	7	7	7	6	7	7	7	7
Respondent 4	5	7	5	6	5	7	5	6	5	5
Respondent 5	4	7	4	6	7	7	4	6	7	4
Respondent 6	3	6	7	6	5	6	7	6	5	3
Respondent 7	2	4	6	5	7	4	6	5	7	2
Respondent 8	5	7	5	7	7	7	5	7	7	5
Respondent 9	7	5	5	2	6	5	5	7	7	4
Respondent 10	6	4	7	4	7	6	5	5	6	7
Respondent 11	5	5	6	5	7	6	4	7	4	6
Respondent 12	7	7	7	7	7	5	5	7	7	5
Respondent 13	5	6	5	5	6	7	7	6	5	5
Respondent 14	4	6	7	4	7	5	6	7	6	5
Respondent 15	7	6	5	3	7	4	6	7	6	4
Respondent 16	6	5	7	2	6	7	6	7	5	5
Respondent 17	5	7	7	5	4	6	5	6	7	7
Respondent 18	5	2	6	7	7	5	7	7	5	6
Respondent 19	5	5	7	4	5	5	2	7	4	6
Respondent 20	2	6	7	7	6	4	7	6	7	6
Respondent 21	6	4	7	7	5	5	6	4	6	5
Respondent 22	5	5	6	6	7	7	7	7	5	7
Respondent 23	7	7	6	4	7	6	4	7	4	4
Respondent 24	5	7	5	5	7	5	5	6	5	5
Respondent 25	4	6	7	7	6	7	7	7	7	7
Respondent 26	7	7	5	6	7	5	6	5	5	6
Respondent 27	6	7	4	6	7	4	6	7	4	6
Respondent 28	5	6	7	6	6	7	6	5	3	6
Respondent 29	5	4	6	5	4	6	5	7	2	5
Respondent 30	5	7	5	7	7	5	7	6	4	7
Respondent 31	5	5	5	2	5	5	7	5	5	6
Respondent 32	7	6	5	5	6	5	6	7	7	7
Respondent 33	7	7	6	4	7	4	7	5	6	5
Respondent 34	6	7	5	5	6	5	7	4	6	7
Respondent 35	6	6	7	7	7	7	6	7	6	5
Respondent 36	5	7	5	6	5	5	4	6	5	7
Respondent 37	7	7	4	6	7	4	7	5	7	7
Respondent 38	5	6	7	6	5	3	5	7	6	4
Respondent 39	4	4	6	5	7	2	6	7	5	5
Respondent 40	7	7	5	7	7	5	7	6	7	7

Respondent 41	6	5	5	2	6	7	7	7	5	6
Respondent 42	5	6	5	5	7	4	6	7	4	6
Respondent 43	5	7	6	4	7	4	7	6	7	6
Respondent 44	4	7	5	5	6	5	7	4	6	5
Respondent 45	5	6	7	7	7	7	7	7	5	7
Respondent 46	7	7	7	7	5	6	5	5	6	7
Respondent 47	6	7	4	6	7	4	6	7	4	6
Respondent 48	5	6	7	6	6	7	6	5	3	6
Respondent 49	5	4	6	5	4	6	5	7	2	5
Respondent 50	5	7	5	7	7	5	7	6	4	7
Respondent 51	5	5	5	2	5	5	7	5	5	6
Respondent 52	7	6	5	5	6	5	6	7	7	7
Respondent 53	7	7	6	4	7	4	7	5	6	5
Respondent 54	6	7	5	5	6	5	7	4	6	7
Respondent 55	6	6	7	7	7	7	6	7	6	5
Respondent 56	5	7	5	6	5	5	4	6	5	7
Respondent 57	7	7	4	6	7	4	7	5	7	7
Respondent 58	5	6	7	6	5	3	5	7	6	4
Respondent 59	4	4	6	5	7	2	6	7	5	5
Respondent 60	7	7	5	7	7	5	7	6	7	7
Respondent 61	6	5	5	2	6	7	7	7	5	6
Respondent 62	5	6	5	5	7	4	6	7	4	6
Respondent 63	5	7	6	4	7	4	7	6	7	6
Respondent 64	4	7	5	5	6	5	7	4	6	5
Respondent 65	6	7	4	6	7	4	6	7	4	6
Respondent 66	5	6	7	6	6	7	6	5	3	6
Respondent 67	5	4	6	5	4	6	5	7	2	5
Respondent 68	5	7	5	7	7	5	7	6	4	7
Respondent 69	5	5	5	2	5	5	7	5	5	6
Respondent 70	7	6	5	5	6	5	6	7	7	7
Respondent 71	7	7	6	4	7	4	7	5	6	5
Respondent 72	6	7	5	5	6	5	7	4	6	7
Respondent 73	6	6	7	7	7	7	6	7	6	5
Respondent 74	5	7	5	6	5	5	4	6	5	7
Respondent 75	7	7	4	6	7	4	7	5	7	7
Respondent 76	5	6	7	6	5	3	5	7	6	4
Respondent 77	4	4	6	5	7	2	6	7	5	5
Respondent 78	7	7	5	7	7	5	7	6	7	7
Respondent 79	6	5	5	2	6	7	7	7	5	6
Respondent 80	5	6	5	5	7	4	6	7	4	6
Respondent 81	5	7	6	4	7	4	7	6	7	6
Respondent 82	4	7	5	5	6	5	7	4	6	5
Respondent 83	5	6	7	7	7	7	7	7	5	7
Respondent 84	5	6	5	5	7	4	6	7	4	6

Respondent 85	5	7	6	4	7	4	7	6	7	6
Respondent 86	4	7	5	5	6	5	7	4	6	5
Respondent 87	5	6	7	7	7	7	7	7	5	7
Respondent 88	6	7	4	6	7	4	6	7	4	6
Respondent 89	5	6	7	6	6	7	6	5	3	6
Respondent 90	5	4	6	5	4	6	5	7	2	5
Respondent 91	5	7	5	7	7	5	7	6	4	7
Respondent 92	5	5	5	2	5	5	7	5	5	6
Respondent 93	7	6	5	5	6	5	6	7	7	7
Respondent 94	7	7	6	4	7	4	7	5	6	5
Respondent 95	6	7	5	5	6	5	7	4	6	7
Respondent 96	6	6	7	7	7	7	6	7	6	5
Respondent 97	5	7	5	6	5	5	4	6	5	7
Respondent 98	7	7	4	6	7	4	7	5	7	7
Respondent 99	5	6	7	6	5	3	5	7	6	4
Respondent 100	4	4	6	5	7	2	6	7	5	5
Respondent 101	7	7	5	7	7	5	7	6	7	7
Respondent 102	6	5	5	2	6	7	7	7	5	6
Respondent 103	5	6	5	5	7	4	6	7	4	6
Respondent 104	5	7	6	4	7	4	7	6	7	6
Respondent 105	4	7	5	5	6	5	7	4	6	5
Respondent 106	5	6	7	7	7	7	7	7	5	7
Respondent 107	5	4	6	5	4	6	5	7	2	5
Respondent 108	5	7	5	7	7	5	7	6	4	7
Respondent 109	5	5	5	2	5	5	7	5	5	6
Respondent 110	7	6	5	5	6	5	6	7	7	7
Respondent 111	7	7	6	4	7	4	7	5	6	5
Respondent 112	6	7	5	5	6	5	7	4	6	7
Respondent 113	6	6	7	7	7	7	6	7	6	5
Respondent 114	5	7	5	6	5	5	4	6	5	7
Respondent 115	7	7	4	6	7	4	7	5	7	7
Respondent 116	5	6	7	6	5	3	5	7	6	4
Respondent 117	4	4	6	5	7	2	6	7	5	5
Respondent 118	7	7	5	7	7	5	7	6	7	7
Respondent 119	6	5	5	2	6	7	7	7	5	6
Respondent 120	5	6	5	5	7	4	6	7	4	6
Respondent 121	5	7	6	4	7	4	7	6	7	6
Respondent 122	4	7	5	5	6	5	7	4	6	5
Respondent 123	6	7	4	6	7	4	6	7	4	6
Respondent 124	5	6	7	6	6	7	6	5	3	6
Respondent 125	5	4	6	5	4	6	5	7	2	5
Respondent 126	5	7	5	7	7	5	7	6	4	7
Respondent 127	5	5	5	2	5	5	7	5	5	6
Respondent 128	7	6	5	5	6	5	6	7	7	7

Respondent 129	7	7	6	4	7	4	7	5	6	5
Respondent 130	6	7	5	5	6	5	7	4	6	7
Respondent 131	6	6	7	7	7	7	6	7	6	5
Respondent 132	5	7	5	6	5	5	4	6	5	7
Respondent 133	7	7	4	6	7	4	7	5	7	7
Respondent 134	5	6	7	6	5	3	5	7	6	4
Respondent 135	4	4	6	5	7	2	6	7	5	5
Respondent 136	7	7	5	7	7	5	7	6	7	7
Respondent 137	6	5	5	2	6	7	7	7	5	6
Respondent 138	5	6	5	5	7	4	6	7	4	6
Respondent 139	5	7	6	4	7	4	7	6	7	6
Respondent 140	4	7	5	5	6	5	7	4	6	5
Respondent 141	5	6	7	7	7	7	7	7	5	7
Respondent 142	5	5	5	2	5	5	7	5	5	6
Respondent 143	7	6	5	5	6	5	6	7	7	7
Respondent 144	7	7	6	4	7	4	7	5	6	5
Respondent 145	6	7	5	5	6	5	7	4	6	7
Respondent 146	6	6	7	7	7	7	6	7	6	5
Respondent 147	5	7	5	6	5	5	4	6	5	7
Respondent 148	7	7	4	6	7	4	7	5	7	7
Respondent 149	5	6	7	6	5	3	5	7	6	4
Respondent 150	4	4	6	5	7	2	6	7	5	5
Respondent 151	7	7	5	7	7	5	7	6	7	7
Respondent 152	6	7	4	6	7	4	6	7	4	6
Respondent 153	5	6	7	6	6	7	6	5	3	6
Respondent 154	5	4	6	5	4	6	5	7	2	5
Respondent 155	5	7	5	7	7	5	7	6	4	7
Respondent 156	5	5	5	2	5	5	7	5	5	6
Respondent 157	7	6	5	5	6	5	6	7	7	7
Respondent 158	7	7	6	4	7	4	7	5	6	5
Respondent 159	6	7	5	5	6	5	7	4	6	7
Respondent 160	6	6	7	7	7	7	6	7	6	5
Respondent 161	5	7	5	6	5	5	4	6	5	7
Respondent 162	7	7	4	6	7	4	7	5	7	7
Respondent 163	5	6	7	6	5	3	5	7	6	4
Respondent 164	4	4	6	5	7	2	6	7	5	5
Respondent 165	7	7	5	7	7	5	7	6	7	7
Respondent 166	6	5	5	2	6	7	7	7	5	6
Respondent 167	5	6	5	5	7	4	6	7	4	6
Respondent 168	5	7	6	4	7	4	7	6	7	6
Respondent 169	4	7	5	5	6	5	7	4	6	5
Respondent 170	5	6	7	7	7	7	7	7	5	7
Respondent 171	6	7	5	5	6	5	7	4	6	7
Respondent 172	6	6	7	7	7	7	6	7	6	5

Respondent 173	5	7	5	6	5	5	4	6	5	7
Respondent 174	7	7	4	6	7	4	7	5	7	7
Respondent 175	5	6	7	6	5	3	5	7	6	4
Respondent 176	6	7	4	6	7	4	6	7	4	6
Respondent 177	5	6	7	6	6	7	6	5	3	6
Respondent 178	5	4	6	5	4	6	5	7	2	5
Respondent 179	5	7	5	7	7	5	7	6	4	7
Respondent 180	5	5	5	2	5	5	7	5	5	6
Respondent 181	7	6	5	5	6	5	6	7	7	7
Respondent 182	7	7	6	4	7	4	7	5	6	5
Respondent 183	6	7	5	5	6	5	7	4	6	7
Respondent 184	6	6	7	7	7	7	6	7	6	5
Respondent 185	5	7	5	6	5	5	4	6	5	7
Respondent 186	7	7	4	6	7	4	7	5	7	7
Respondent 187	5	6	7	6	5	3	5	7	6	4
Respondent 188	6	7	4	6	7	4	6	7	4	6
Respondent 189	5	6	7	6	6	7	6	5	3	6
Respondent 190	5	4	6	5	4	6	5	7	2	5
Respondent 191	5	7	5	7	7	5	7	6	4	7
Respondent 192	5	5	5	2	5	5	7	5	5	6
Respondent 193	7	6	5	5	6	5	6	7	7	7
Respondent 194	7	7	6	4	7	4	7	5	6	5
Respondent 195	6	7	5	5	6	5	7	4	6	7
Respondent 196	6	6	7	7	7	7	6	7	6	5
Respondent 197	5	7	5	6	5	5	4	6	5	7
Respondent 198	7	7	4	6	7	4	7	5	7	7
Respondent 199	5	6	7	6	5	3	5	7	6	4
Respondent 200	4	4	6	5	7	2	6	7	5	5
Respondent 201	7	7	5	7	7	5	7	6	7	7
Respondent 202	6	5	5	2	6	7	7	7	5	6
Respondent 203	5	6	5	5	7	4	6	7	4	6
Respondent 204	5	7	6	4	7	4	7	6	7	6
Respondent 205	4	7	5	5	6	5	7	4	6	5
Respondent 206	5	6	7	7	7	7	7	7	5	7
Respondent 207	5	6	7	6	5	3	5	7	6	4
Respondent 208	4	4	6	5	7	2	6	7	5	5
Respondent 209	7	7	5	7	7	5	7	6	7	7
Respondent 210	6	7	4	6	7	4	6	7	4	6
Respondent 211	5	6	7	6	6	7	6	5	3	6
Respondent 212	5	4	6	5	4	6	5	7	2	5
Respondent 213	5	7	5	7	7	5	7	6	4	7
Respondent 214	5	5	5	2	5	5	7	5	5	6
Respondent 215	7	6	5	5	6	5	6	7	7	7
Respondent 216	7	7	6	4	7	4	7	5	6	5

Respondent 217	6	7	5	5	6	5	7	4	6	7
Respondent 218	6	6	7	7	7	7	6	7	6	5
Respondent 219	5	7	5	6	5	5	4	6	5	7
Respondent 220	7	7	4	6	7	4	7	5	7	7
Respondent 221	5	6	7	6	5	3	5	7	6	4
Respondent 222	4	4	6	5	7	2	6	7	5	5
Respondent 223	7	7	5	7	7	5	7	6	7	7
Respondent 224	6	5	5	2	6	7	7	7	5	6
Respondent 225	5	6	5	5	7	4	6	7	4	6
Respondent 226	5	7	6	4	7	4	7	6	7	6
Respondent 227	4	7	5	5	6	5	7	4	6	5
Respondent 228	5	6	7	7	7	7	7	7	5	7
Respondent 229	5	7	5	6	5	5	4	6	5	7
Respondent 230	7	7	4	6	7	4	7	5	7	7
Respondent 231	5	6	7	6	5	3	5	7	6	4
Respondent 232	4	4	6	5	7	2	6	7	5	5
Respondent 233	7	7	5	7	7	5	7	6	7	7
Respondent 234	6	7	4	6	7	4	6	7	4	6
Respondent 235	5	6	7	6	6	7	6	5	3	6
Respondent 236	5	4	6	5	4	6	5	7	2	5
Respondent 237	5	7	5	7	7	5	7	6	4	7
Respondent 238	5	5	5	2	5	5	7	5	5	6
Respondent 239	7	6	5	5	6	5	6	7	7	7
Respondent 240	7	7	6	4	7	4	7	5	6	5
Respondent 241	6	7	5	5	6	5	7	4	6	7
Respondent 242	6	6	7	7	7	7	6	7	6	5
Respondent 243	5	7	5	6	5	5	4	6	5	7
Respondent 244	7	7	4	6	7	4	7	5	7	7
Respondent 245	5	6	7	6	5	3	5	7	6	4
Respondent 246	4	4	6	5	7	2	6	7	5	5
Respondent 247	7	7	5	7	7	5	7	6	7	7
Respondent 248	6	5	5	2	6	7	7	7	5	6
Respondent 249	5	6	5	5	7	4	6	7	4	6
Respondent 250	5	7	6	4	7	4	7	6	7	6
Respondent 251	4	7	5	5	6	5	7	4	6	5
Respondent 252	5	6	7	7	7	7	7	7	5	7
Respondent 253	5	4	6	5	4	6	5	7	2	5
Respondent 254	5	7	5	7	7	5	7	6	4	7
Respondent 255	5	5	5	2	5	5	7	5	5	6
Respondent 256	7	6	5	5	6	5	6	7	7	7
Respondent 257	7	7	6	4	7	4	7	5	6	5
Respondent 258	6	7	5	5	6	5	7	4	6	7
Respondent 259	6	6	7	7	7	7	6	7	6	5
Respondent 260	6	7	4	6	7	4	6	7	4	6

Respondent 261	5	6	7	6	6	7	6	5	3	6
Respondent 262	5	4	6	5	4	6	5	7	2	5
Respondent 263	5	7	5	7	7	5	7	6	4	7
Respondent 264	5	5	5	2	5	5	7	5	5	6
Respondent 265	7	6	5	5	6	5	6	7	7	7
Respondent 266	7	7	6	4	7	4	7	5	6	5
Respondent 267	6	7	5	5	6	5	7	4	6	7
Respondent 268	6	6	7	7	7	7	6	7	6	5
Respondent 269	5	7	5	6	5	5	4	6	5	7
Respondent 270	7	7	4	6	7	4	7	5	7	7
Respondent 271	5	6	7	6	5	3	5	7	6	4
Respondent 272	4	4	6	5	7	2	6	7	5	5
Respondent 273	7	7	5	7	7	5	7	6	7	7
Respondent 274	6	5	5	2	6	7	7	7	5	6
Respondent 275	5	6	5	5	7	4	6	7	4	6
Respondent 276	5	7	6	4	7	4	7	6	7	6
Respondent 277	4	7	5	5	6	5	7	4	6	5
Respondent 278	5	6	7	7	7	7	7	7	5	7
Respondent 279	5	7	5	7	7	7	5	7	7	5
Respondent 280	7	5	5	2	6	5	5	7	7	4
Respondent 281	6	4	7	4	7	6	5	5	6	7
Respondent 282	5	5	6	5	7	6	4	7	4	6
Respondent 283	7	7	7	7	7	5	5	7	7	5
Respondent 284	5	6	5	5	6	7	7	6	5	5
Respondent 285	4	6	7	4	7	5	6	7	6	5
Respondent 286	7	6	5	3	7	4	6	7	6	4
Respondent 287	6	5	7	2	6	7	6	7	5	5
Respondent 288	5	7	7	5	4	6	5	6	7	7
Respondent 289	5	2	6	7	7	5	7	7	5	6
Respondent 290	4	7	6	4	7	7	6	4	7	4
Respondent 291	5	7	5	5	6	7	5	5	6	5
Respondent 292	7	6	7	7	7	6	7	7	7	7
Respondent 293	5	7	5	6	5	7	5	6	5	5
Respondent 294	4	7	4	6	7	7	4	6	7	4
Respondent 295	3	6	7	6	5	6	7	6	5	3
Respondent 296	2	4	6	5	7	4	6	5	7	2
Respondent 297	5	7	5	7	7	7	5	7	7	5
Respondent 298	7	5	5	2	6	5	5	7	7	4
Respondent 299	6	4	7	4	7	6	5	5	6	7
Respondent 300	5	5	6	5	7	6	4	7	4	6
Respondent 301	7	7	7	7	7	5	5	7	7	5
Respondent 302	5	6	5	5	6	7	7	6	5	5
Respondent 303	4	6	7	4	7	5	6	7	6	5
Respondent 304	7	6	5	3	7	4	6	7	6	4

Respondent 305	6	5	7	2	6	7	6	7	5	5
Respondent 306	5	7	7	5	4	6	5	6	7	7
Respondent 307	5	2	6	7	7	5	7	7	5	6
Respondent 308	5	5	7	4	5	5	2	7	4	6
Respondent 309	2	6	7	7	6	4	7	6	7	6
Respondent 310	6	4	7	7	5	5	6	4	6	5
Respondent 311	5	5	6	6	7	7	7	7	5	7
Respondent 312	7	7	7	7	5	6	5	5	5	2
Respondent 313	7	6	7	7	7	6	7	7	7	7
Respondent 314	5	7	5	6	5	7	5	6	5	5
Respondent 315	4	7	4	6	7	7	4	6	7	4
Respondent 316	3	6	7	6	5	6	7	6	5	3
Respondent 317	2	4	6	5	7	4	6	5	7	2
Respondent 318	5	7	5	7	7	7	5	7	7	5
Respondent 319	7	5	5	2	6	5	5	7	7	4
Respondent 320	6	4	7	4	7	6	5	5	6	7
Respondent 321	5	5	6	5	7	6	4	7	4	6
Respondent 322	7	7	7	7	7	5	5	7	7	5
Respondent 323	5	6	5	5	6	7	7	6	5	5
Respondent 324	4	6	7	4	7	5	6	7	6	5
Respondent 325	7	6	5	3	7	4	6	7	6	4
Respondent 326	6	5	7	2	6	7	6	7	5	5
Respondent 327	5	7	7	5	4	6	5	6	7	7
Respondent 328	5	2	6	7	7	5	7	7	5	6
Respondent 329	5	5	7	4	5	5	2	7	4	6
Respondent 330	2	6	7	7	6	4	7	6	7	6
Respondent 331	6	4	7	7	5	5	6	4	6	5
Respondent 332	5	5	6	6	7	7	7	7	5	7
Respondent 333	7	7	7	7	5	6	5	5	5	2
Respondent 334	5	7	5	7	7	7	5	7	7	5
Respondent 335	7	5	5	2	6	5	5	7	7	4
Respondent 336	6	4	7	4	7	6	5	5	6	7
Respondent 337	5	5	6	5	7	6	4	7	4	6
Respondent 338	7	7	7	7	7	5	5	7	7	5
Respondent 339	5	7	5	7	7	7	5	7	7	5
Respondent 340	7	5	5	2	6	5	5	7	7	4
Respondent 341	6	4	7	4	7	6	5	5	6	7
Respondent 342	5	5	6	5	7	6	4	7	4	6
Respondent 343	7	7	7	7	7	5	5	7	7	5
Respondent 344	5	6	5	5	6	7	7	6	5	5
Respondent 345	4	6	7	4	7	5	6	7	6	5
Respondent 346	7	6	5	3	7	4	6	7	6	4
